# Integrated computational and experimental analysis explores FOLH1 expression patterns across cancers and nominates melatonin as a potential modulator in prostate cancer models

**DOI:** 10.1371/journal.pcbi.1014315

**Published:** 2026-05-22

**Authors:** Rui Zhang, Junyu Zhou, Sihan Dong, Guoquan Liu, Xunbin Wei

**Affiliations:** 1 Institute of Medical Technology, Peking University Health Science Center, Beijing, China; 2 Institute of Advanced Clinical Medicine, Peking University, Beijing, China; 3 Department of Biomedical Engineering, Peking University, Beijing, China; Peking University Health Science Center, Institute of Medical Technology, CANADA

## Abstract

**Background:**

Growing evidence indicates that Folate Hydrolase 1 (FOLH1, also known as prostate-specific membrane antigen, PSMA) is aberrantly expressed across multiple malignancies, particularly showing significant upregulation in prostate cancer. However, systematic investigations into its pan-cancer expression patterns, immunomodulatory roles, and immune cell infiltration remain limited. The potential role of FOLH1 in prostate cancer is also not fully elucidated.

**Methods:**

We analyzed FOLH1 mRNA expression, prognostic relevance, and immune infiltration across multiple malignancies, with a particular focus on prostate cancer. A machine learning (ML) workflow incorporating a deep learning model was developed to screen the therapeutic potential of drugs targeting FOLH1. The therapeutic potential of these candidates was validated through in vitro cellular assays and nude mouse xenograft models.

**Results:**

FOLH1 expression was significantly altered in 27 cancer types and showed cancer-specific immune correlations. Our AI platform identified melatonin as a computationally predicted FOLH1-interacting candidate. *In vitro* and *in vivo* experiments demonstrated that melatonin suppresses FOLH1 expression in a concentration-dependent manner, inhibits invasive and migratory capacities, and restricts tumor growth under physiological circadian melatonin levels.

**Conclusion:**

This study highlights FOLH1’s pan-cancer expression patterns and nominates melatonin as an exploratory therapeutic candidate for prostate cancer requiring further mechanistic validation. Our integrated computational-experimental framework highlights the promise of AI-driven drug discovery in oncology, while emphasizing the need for further mechanistic validation.

## Author summary

Cancer remains a leading health threat worldwide, making the discovery of new treatment targets essential. In this study, we investigated a specific protein that is often highly active in prostate cancer and several other tumor types. By analyzing large public medical databases, we found that this protein’s presence is linked to tumor behavior and how the immune system responds across various cancers. To accelerate the search for potential new treatments, we built an artificial intelligence computer program to screen thousands of existing medications. Our computer model identified melatonin, a common natural hormone best known for regulating sleep, as a strong candidate to interact with this cancer-related protein. We tested this computationally generated idea in the laboratory and found that treating prostate cancer cells with melatonin reduced the protein’s levels and slowed the cancer’s ability to spread. Furthermore, disrupting the natural sleep-wake cycles in animal models, which lowers their natural melatonin, caused tumors to grow faster. Our findings demonstrate how computer programs might help identify new uses for existing drugs and suggest that melatonin could be explored further as a potential supportive approach in prostate cancer research.

## 1. Introduction

Cancer has emerged as a major public health threat globally. According to the latest global cancer statistics, approximately 20 million new cancer cases were diagnosed in 2022, accompanied by 9.7 million cancer-related deaths [1]. Advances in cancer research and mechanistic understanding of oncogenesis have driven significant progress in therapeutic modalities, including immunotherapy, targeted therapies, and radiotherapy. Notably, the development of immunotherapy has revolutionized cancer treatment paradigms, establishing itself as a first-line intervention for select malignancies [2,3]. The rapid expansion of genomic databases such as The Cancer Genome Atlas (TCGA) and Gene Expression Omnibus (GEO) now enables systematic identification of prognostic biomarkers and molecular targets across pan-cancer analyses [4]. Despite these advances, cancer remains one of the leading causes of morbidity and mortality worldwide, with significant heterogeneity in treatment responses across different tumor types and patient populations. This variability underscores the critical need for continued exploration of potential biomarkers and therapeutic targets that can improve patient stratification and treatment outcomes across diverse cancer types [5]. Moreover, the integration of multi-omics data and computational approaches has accelerated the discovery and validation of potential cancer biomarkers, offering unprecedented opportunities for precision oncology applications [6].

FOLH1 encodes a type II transmembrane glycoprotein belonging to the M28 peptidase family, it has both folate hydrolase and N-acetylated-alpha-linked-acidic dipeptidase (NAALADase) activity [7]. In the gut, it is involved in the absorption of folate, and in the brain, by hydrolyzing N-acetyl-l-aspartyl-l-glutamate (NAAG), it regulates the transmission of excitatory neurotransmitters [8]. In recent years, a growing number of studies have found an association between FOLH1 and the progression of several tumors, including kidney cancer, Merkel cell carcinoma (MCC), endometrial carcinoma [9–11]. In prostate cancer, FOLH1 expression is significantly higher in prostate cancer compared to normal tissue, making it a promising target for diagnosis, imaging, and therapy, in addition, FOLH1 overexpression is linked to cancer progression, metastasis, and androgen independence [12,13]. At present, a variety of drugs targeting FOLH1 have been used in the imaging and treatment of tumors, and have achieved good results [14]. FOLH1 is increasingly recognized for its dual role as both a diagnostic biomarker and therapeutic target in oncology. The glycoprotein’s distinctive overexpression pattern in tumor-associated neovasculature beyond prostate cancer suggests broader implications across multiple malignancies [10,15,16]. Its internalization properties upon ligand binding make it attractive for targeted drug delivery approaches and radioligand therapies. Recent clinical successes with FOLH1-targeted radioligand therapies, such as 177Lu-FOLH1–617, have demonstrated remarkable efficacy in metastatic castration-resistant prostate cancer, highlighting the therapeutic potential of FOLH1 targeting strategies [17]. Furthermore, the development of FOLH1-targeted antibody-drug conjugates and small molecule inhibitors represents an expanding frontier in precision oncology that warrants comprehensive investigation across cancer types [18].

While these studies provide support for the possibility that FOLH1 may influence the progression of a range of cancers, there is currently a lack of pan-cancerous studies evaluating FOLH1’s role. The advancement of computational technologies, particularly artificial intelligence (AI) and machine learning, has enabled more precise and efficient screening of potential therapeutic agents through integration of molecular representation techniques, advanced feature extraction methodologies, and sophisticated predictive modeling architectures [19]. Current state of computational approaches in drug discovery have evolved significantly, incorporating sophisticated methodologies that enhance accuracy and efficiency. Specifically, integrative frameworks combining machine learning with molecular informatics have demonstrated remarkable efficacy in compound identification and optimization. The implementation of Morgan fingerprints, circular topological descriptors that capture molecular substructures, has proven particularly valuable for representing chemical entities in high-dimensional feature spaces, facilitating more precise similarity assessments and activity predictions [20]. Furthermore, ensemble learning paradigms, including Extra Trees regressors and gradient-boosting frameworks such as LightGBM, have substantially improved predictive performance by mitigating model variance and enhancing generalizability across diverse chemical spaces [21]. Concurrently, the advent of deep neural architectures capable of processing multiple molecular representation modalities, ranging from two-dimensional (2D) fingerprints to three-dimensional (3D) conformational descriptors and pharmacophore features, has revolutionized virtual screening campaigns through their capacity to capture complex structure-activity relationships [22]. Our study leverages these established computational strategies within a cohesive methodological framework, thereby exemplifying the transformative potential of integrative computational approaches in contemporary pharmacological research and target-specific therapeutic discovery. Recent advancements in computational drug discovery have further refined our ability to identify therapeutic candidates targeting specific molecular entities like FOLH1. The integration of structural biology insights with deep learning architectures has enabled more accurate prediction of protein-ligand interactions and binding affinities [23]. Additionally, the application of transfer learning approaches and graph neural networks has significantly improved the representation of complex molecular structures and their physicochemical properties, leading to enhanced predictive performance in virtual screening campaigns [24]. These computational advances, coupled with the growing availability of structural data for FOLH1, present an unprecedented opportunity to accelerate the discovery of novel therapeutic agents through in silico approaches before experimental validation.

In this paper, we systematically analyzed FOLH1 expression patterns across pan-cancer malignancies through interrogation of TCGA, GTEx, UCSC Xena, and HPA databases, with extended evaluation of its correlations with immune cell infiltration/immune-related genes, particularly in prostate cancer. Our methodology involved:1) Development of a comprehensive ML workflow integrating multiple molecular representation techniques; 2) Creation of ChemFusionSimilarity - a deep learning model enabling accurate molecular similarity predictions across heterogeneous feature spaces; 3) Implementation of feature selection and clustering methodologies to establish drug activity classification criteria. This computational pipeline identified melatonin as a candidate compound with predicted binding potential to FOLH1. Subsequent validation through in vitro cellular assays and in vivo xenograft models confirmed melatonin’s capacity to suppress FOLH1 expression. Therefore, we hypothesized that FOLH1 plays a critical role in prostate cancer progression and immune modulation, and that modulating FOLH1 expression, potentially with computationally identified agents like melatonin, could offer therapeutic benefits, possibly influenced by circadian rhythms. The significance of our study extends beyond the identification of melatonin as a potential FOLH1-targeting agent. By establishing a comprehensive pan-cancer profile of FOLH1 expression and its relationship with clinical outcomes, immune infiltration patterns, and molecular signatures, we provide a foundation for future investigations into FOLH1’s role across diverse malignancies. Furthermore, our integrated computational-experimental approach demonstrates the power of leveraging AI/ML methodologies in conjunction with traditional experimental validation to accelerate biomarker discovery and drug repurposing efforts. The findings presented herein not only expand our understanding of FOLH1 biology in cancer but also suggest potential avenues for further investigation into melatonin repurposing of melatonin, a well-characterized drug compound with an established safety profile, potentially expediting clinical translation.

## 2. Materials and methods

### 2.2. Ethics statement

All animal experimental procedures were approved by the Institutional Animal Care and Use Committee of Peking University Health Science Center. All experiments were performed in accordance with relevant guidelines and regulations.

## 3. Bioinformatic analysis

### 3.1. mRNA expression analysis of FOLH1

To compare the expression levels of FOLH1 between normal tissues and pan-cancer tissues in TCGA database [25], the TIMER 2.0 online tool (http://timer.comp-genomics.org/) was utilized. We downloaded the uniformly standardized pan-cancer dataset from the UCSC database (https://xenabrowser.net/), extracted FOLH1 gene expression data across samples, and excluded cancer types with fewer than three samples. Using R software (version 3.6.4), the expression differences between normal and tumor samples were calculated for each cancer type. The unpaired Wilcoxon Rank Sum and Signed Rank Tests were applied for significance analysis (*P* < 0.05 was considered statistically significant), and results were visualized using the Sangerbox online tool (http://sangerbox.com/index.html). Meanwhile, the single-cell distribution of FOLH1 was analyzed using the HPA database.

### 3.2. Expression correlation analysis

The standardized TCGA Pan-Cancer dataset was downloaded from UCSC. After data filtering and matching, the correlation between FOLH1 expression and patient prognosis under different stratification criteria was analyzed. Pearson correlation coefficients were calculated for each cancer type using R software.

### 3.3. Correlation of FOLH1 expression with immune cell infiltration and immune-related genes

The online platform ASSISTANT for Clinical Bioinformatics (https://www.aclbi.com/static/index.html) was employed to assess tumor immune cell infiltration levels using six algorithms: TIMER, CIBERSORT, xCell, MCP-Counter, EPIC, and QUANTISEQ. The correlation between FOLH1 expression and infiltration levels of immune cell types, including B cells, CD8^+^ T cells, CD4^+^ T cells, monocytes, neutrophils, MDSCs, mast cells, macrophages, cancer-associated fibroblasts, Tregs, myeloid dendritic cells, and NK cells, was analyzed. Additionally, SangerBox was used to evaluate Pearson correlations between FOLH1 and 150 immune pathway marker genes across five categories: chemokines (41), receptors (18), MHC molecules (21), immunoinhibitors (24), and immunostimulators (46).

### 3.4. FOLH1 analysis in prostate cancer

Given the pronounced upregulation of FOLH1 in prostate cancer, immunohistochemical staining images of FOLH1 in normal and tumor tissues were obtained from the Human Protein Atlas. Prostate adenocarcinoma (PRAD) data and clinical information were downloaded from TCGA. R packages (ggplot2 and immunedeconv (v2.1.3)) in R software were used to analyze FOLH1 expression in lymph node metastasis subgroups and corresponding immune infiltration. The corresponding single-cell data in.h5 format and annotation results were downloaded from TISCH [26]. The R packages MAESTRO and Seurat (v4.4.0) were utilized to process and analyze the single-cell data. Subsequently, the cells were re-clustered using the t-SNE method. When utilizing Seurat for t-SNE clustering, we meticulously debug and configure the key parameters. During the data preprocessing stage, we optimized the data normalization method to align with the algorithmic characteristics of MAESTRO, thereby enhancing its compatibility with t-SNE clustering and improving the overall clustering results. For cell type annotation, we first identified cell-specific markers based on authoritative literature and databases. For instance, in the context of T cells, we focused on key markers such as CD3, CD4, and CD8, while for macrophages, we emphasized markers like CD68 and CD163, fibroblasts (COL1A1 + , COL3A1+), epithelial cells (EPCAM + , KRT8 + , KRT18+) [26]. Cell clusters were validated through comparison with reference signatures from the PanglaoDB and CellMarker databases. Only cells expressing ≥3 canonical markers for a specific cell type with expression levels >1.5-fold compared to other cell types were definitively annotated. The ESTIMATE package (v1.0.13) calculated stromal, immune, and ESTIMATE scores in PRAD, while the psych package (v2.1.6) determined Pearson correlations between FOLH1 expression and immune infiltration scores.

## 4. Computational drug discovery

### 4.1. Data collection and preprocessing in drug compound activity prediction using machine learning

To develop a robust computational framework for identifying drug candidates with therapeutic potential against prostate cancer, we employed a comprehensive machine learning workflow focused on predicting the activity (expressed as pIC_50_) of ligands associated with FOLH1, a target protein linked to prostate cancer. Only a limited number of FOLH1 ligands with quantitative activity annotations were available in public databases (420 compounds), the FOLH1 dataset alone was insufficient for robust pIC_50_ prediction model training. An alternative data expansion strategy was adopted for target selection, we shifted our focus to target proteins exhibiting strong correlations with prostate cancer. These proteins were selected from the ProteinAtlas database [27], a large public repository of human protein data, were selected based on rigorous multi-dimensional criteria: (1) Differential expression in prostate cancer versus normal prostate tissue (fold change ≥ 2.0, p < 0.01); (2) Significant association with clinical outcomes (survival, recurrence, or metastasis) based on Kaplan-Meier analyses and multivariate Cox regression models to identify proteins with prognostic relevance; (3) Confirmed protein-level overexpression by immunohistochemistry in at least 70% of examined prostate cancer tissue samples, providing visual validation of expression patterns across tumor grades and stages; (4) Druggability potential based on structural features and well-characterized structural properties amenable to computational drug discovery, including the presence of defined binding pockets, surface accessibility, and functional domains that can interact with small molecules. These proteins were further filtered based on their protein evidence scores derived from multiple independent sources, categorized into four levels: (1) evidence at the protein level, (2) evidence at the transcript level, (3) no evidence, or (4) not analyzed. For this study, we prioritized target proteins with robust evidence (level 1) of association with prostate cancer to ensure biological relevance and increase the translational potential of our computational predictions.

Ligand data for these selected target proteins were aggregated from multiple chemical databases, including ChEMBL [28], PubChem [29], ChemSpider [30], ZINC [31], BindingDB [32], PDBbind [33], and STITCH [34]. Ligand data, stored in a CSV file containing Simplified Molecular Input Line Entry System (SMILES) strings [35] and corresponding IC_50_ values, were imported using the Pandas library. Data quality was ensured by employing the Chem.MolFromSmiles function from the RDKit library [36] to filter out invalid SMILES strings and entries with missing or anomalous IC_50_ values.

### 4.2. Feature extraction and normalization for effective drug compound activity prediction

Molecular fingerprints were generated using Morgan fingerprints via the RDKit library. These fingerprints, based on atomic neighborhood information, efficiently capture the topological structure of each molecule. In parallel, we computed a series of molecular descriptors to characterize the physicochemical properties of the compounds. These descriptors included: MolLogP, the hydrophobicity of the molecule (partition coefficient, LogP); MolWt, molecular weight; NumRotatableBonds, number of rotatable bonds; TPSA, topological polar surface area; NumHAcceptors, number of hydrogen bond acceptors, NumHDonors, number of hydrogen bond donors. The Morgan fingerprints and molecular descriptors were concatenated to construct a comprehensive feature matrix, serving as the input for the machine learning models.

### 4.3. Data partitioning, model construction, and performance evaluation

To assess the generalization performance of our predictive models, 80% of the data were allocated to the training set for model development, while the remaining 20% were reserved as the test set. Our machine learning framework incorporates multiple algorithms, with selection guided by several critical criteria: (1) demonstrated benchmark performance in similar ligand-binding prediction tasks; (2) capacity to efficiently process high-dimensional chemical descriptors; (3) interpretability of feature importance; and (4) computational efficiency during both training and inference phases. The selected models include: Ridge Regression [37], a linear regression model incorporating L2 regularization to minimize overfitting by penalizing large coefficients; Linear Support Vector Regression [38] (Linear SVR), a regression model based on support vector machines with a linear kernel, designed to optimize a margin-based loss function; Extra Trees Regressor [39], an ensemble learning model utilizing randomized decision trees, which enhances generalization through random feature selection and sample subsampling; and LightGBM Regressor [40], a gradient boosting framework optimized for efficiency and scalability, particularly effective for large datasets. Additionally, we extended our model comparison to include five other established methods: XGBoost [41], which implements gradient boosting with regularization techniques to prevent overfitting; Graph Neural Networks (GNNs) [42], which directly operate on molecular graph structures to capture complex structural relationships; Convolutional Neural Networks (CNNs) [43], which extract hierarchical features from molecular representations; and Random Forests (RF) [44], which leverage ensemble decision trees with bootstrap aggregation.

To strengthen the robustness of our machine learning validation protocol, we implemented 5-fold cross-validation for all models, ensuring reliable performance assessment across different data subsets. For comprehensive evaluation, we report multiple performance metrics including accuracy, precision, recall, and F1 score, alongside the standard mean absolute error (MAE), RMSE and coefficient of determination (R²). Robustness testing was performed through perturbation analysis, where we systematically varied data partitioning schemes, feature selection thresholds, and hyperparameter settings to assess model stability under different conditions. Each model was trained on the feature matrix derived from the training set, with hyperparameters tuned through grid search to optimize performance across the selected metrics.

#### 4.3.1. Dataset preparation for ligand-drug interaction prediction.

We constructed a classification model to evaluate the interaction potential between FOLH1 ligands and drug molecules. The FOLH1 ligand dataset was divided into positive and negative subsets to create the training dataset. Positive pairs, labeled as 1, represented ligands with high biological activity, while negative pairs, labeled as 0, denoted ligands with low biological activity. The model was trained to calculate the similarity between ligands and drug molecules, enabling the prediction of new drugs with the potential to modulate FOLH1. The computed pIC_50_ values and their corresponding molecular descriptors were organized into a two-dimensional array, where each row encapsulated the descriptor values for an individual molecule.

#### 4.3.2. Classification of ligand activity.

To facilitate candidate prioritization, we additionally performed a secondary binary classification analysis by discretizing pIC50 values into high-activity and low-activity groups. Ligands were categorized into three activity levels based on predefined pIC_50_ thresholds, aligned with established standards in drug discovery: Low Activity (pIC_50_ < 5), indicating minimal biological activity and limited therapeutic potential; Medium Activity (5 ≤ pIC_50_ ≤ 7), reflecting moderate biological activity that may warrant further optimization to enhance efficacy; High Activity (pIC_50_ > 7), designating potential drug candidates. This threshold was chosen based on the observation that many approved drugs exhibit pIC_50_ values above 7, reflecting sufficient potency for therapeutic applications. In addition, we applied Lipinski’s Rule of Five to evaluate the drug-likeness of the molecules. According to this rule, ideal drug candidates should exhibit a LogP value less than 5, a molecular weight below 500 Da, fewer than 5 hydrogen bond donors, and fewer than 10 hydrogen bond acceptors. We applied the K-means clustering algorithm to the standardized molecular descriptor data. The pairwise relationships among molecular descriptors were visualized using the Pairplot function from the Seaborn library [45].

#### 4.3.3. ChemFusionsimilarity model architecture.

We constructed a deep learning-based model, termed ChemFusionSimilarity (https://github.com/Benjamin-JHou/ChemFusionSimilarity), to predict the similarity between ligands and drug molecules. To evaluate practical utility in virtual screening, we compared the enrichment of high-activity compounds among the top-ranked neighbors retrieved by ChemFusionSimilarity and by Tanimoto similarity. This model integrates multiple molecular representation methods, namely molecular descriptors, Morgan fingerprints, and SELFIES (Self-referencing Embedded Strings) encoding [46], to achieve a comprehensive assessment of molecular similarity. By combining attention mechanisms and feature fusion techniques, ChemFusionSimilarity leverages the complementary strengths of these representations to overcome the limitations inherent in single-representation approaches. Specifically, SELFIES capture the topological structure of molecules, Morgan fingerprints highlight local structural motifs, and molecular descriptors provide macroscopic physicochemical properties. The incorporation of an attention mechanism enables the model to dynamically adjust the importance of each feature type, thereby improving the precision of similarity predictions.

#### 4.3.4. Molecular representation encoders.

The ChemFusionSimilarity model employs three independent encoders to process distinct categories of molecular features. SELFIES Encoder: Input (SELFIES-encoded vectors with a dimensionality of selfies_embed_dim); Encoder Architecture (a linear layer maps the SELFIES vectors into a shared hidden space); SELFIES (a character-based representation, encodes molecular structures into reversible strings, offering a robust method to capture topological information essential for similarity evaluation). Morgan Fingerprint Encoder: Input (Morgan fingerprint vectors/Extended-Connectivity Fingerprints, ECFP with a dimensionality of fp_dim); Encoder Architecture (encoder comprises a linear layer, followed by a ReLU activation function, a Dropout layer, and a Batch Normalization layer). The architecture transforms the fingerprint vectors into the same hidden space as the SELFIES and descriptor encoders, enabling feature integration. Molecular Descriptor Encoder: Input (molecular descriptor vectors with a dimensionality of descriptor_dim); Encoder Architecture (similar to the Morgan fingerprint encoder, this module consists of a linear layer, ReLU activation, Dropout, and BatchNorm layers). It maps the descriptor vectors into the shared hidden space, providing a representation of the molecule’s macroscopic physicochemical properties. To dynamically weigh the contributions of the three feature encoders, the ChemFusionSimilarity model incorporates a multi-head attention mechanism (nn.MultiheadAttention). In this setup, the output of the SELFIES encoder serves as the query, while the outputs of the Morgan fingerprint and molecular descriptor encoders act as keys and values, respectively. This configuration enables the model to prioritize features from the fingerprint and descriptor encoders based on the topological context provided by SELFIES. The attention mechanism generates a weighted feature representation, enhancing the model’s capacity to capture intricate molecular similarities.

Following the attention mechanism, the model fuses the outputs of the three encoders to produce a final similarity prediction. The fusion process begins by concatenating the attention-weighted SELFIES output with the outputs of the Morgan fingerprint and molecular descriptor encoders. This combined feature vector is then processed through a multi-layer perceptron, which maps it to a lower-dimensional space and outputs a similarity score ranging from 0 to 1. This score quantifies the predicted similarity between ligand-drug molecule pairs, integrating diverse molecular characteristics into a unified metric.

#### 4.3.5. Model training and optimization.

The ChemFusionSimilarity model was trained using the Adam optimizer and the Mean Squared Error (MSE) loss function (nn.MSELoss). To optimize the learning rate dynamically based on validation set performance, we employed the ReduceLROnPlateau scheduler. During each training epoch, the model underwent forward propagation, loss computation, backpropagation, and parameter updates on the training dataset. Model performance was assessed on a separate validation set, with validation loss monitored to ensure convergence and prevent overfitting. To further enhance training stability and mitigate overfitting, Dropout and Batch Normalization techniques were applied within both the encoders and the feature fusion layer. As a benchmark for molecular similarity, we incorporated the Tanimoto similarity coefficient, a widely used metric for comparing molecular fingerprints. For each ligand-drug pair, the Tanimoto similarity [47] was calculated and combined with the ChemFusionSimilarity scores into a unified dataframe.

### 4.4. Drug data acquisition and preprocessing

To initiate the drug screening process, we downloaded drug-related data from DrugBank [48], a widely recognized repository of approved and investigational pharmaceuticals. Subsequently, SMILES strings, which provide a text-based representation of molecular structures, were retrieved for drug compounds from multiple chemical databases, including PubChem, ChemSpider, and ChEMBL. To eliminate structurally redundant compounds and prioritize molecular diversity, we applied the Uniform Manifold Approximation and Projection (UMAP) algorithm for dimensionality reduction. Following this screening, the dataset was refined to candidate drugs. After the initial screening, similarity calculations were performed using ChemFusionSimilarity model architecture.

### 4.5. Molecular docking simulations with AutoDock Vina

The drug molecule exhibiting the highest similarity score to the FOLH1 ligand was selected for molecular docking studies to assess its potential binding affinity with the FOLH1 protein, using AutoDock Vina [49], a robust and widely utilized tool for predicting ligand-protein binding poses and affinities. The docking workflow encompassed the following key stages: (1) Molecular Structure Preparation: Ligand Optimization, initial 3D conformations of the selected drug molecules were generated using the MMFF94 force field, a well-established method for molecular mechanics calculations. These structures underwent energy minimization via the conjugate gradient method, with a convergence threshold of 0.01 kcal/mol. For each drug, the conformer with the lowest energy was chosen as the starting structure for docking, ensuring an energetically favorable configuration. (2) Protein Preparation: The FOLH1 protein structure (PDB: 3LUT) was obtained from the Protein Data Bank (PDB) [50] and processed to optimize its suitability for docking. This preparation involved removing crystallographic water molecules, adding hydrogen atoms, and adjusting the charge distribution using Discovery Studio Visualizer [50] (version 19.1), thereby ensuring compatibility with the docking algorithm. (3) Docking Procedure, a semi-flexible docking strategy was employed, wherein the ligand was allowed conformational flexibility while the protein receptor remained rigid.

The docking site was defined based on the binding pocket of the co-crystallized ligand within the FOLH1 receptor, ensuring biological relevance. AutoDock Vina was configured with a grid box centered at (x = 15.23, y = -8.45, z = 22.10) Å with dimensions of 20 × 20 × 20 Å, an exhaustiveness value of 16, and 9 binding poses were generated for each ligand. Docking outcomes were visualized using Discovery Studio Visualizer and UCSF Chimera [51], allowing for an in-depth examination of ligand-protein interactions. Specific criteria were established to identify hydrogen bonds, including a maximum donor-acceptor distance of 2.50 Å (e.g., O-H), a minimum angle of 120°, and consideration of bimolecular base-induced 1,2-elimination (E2) reactions within the crystallographic context. Binding affinities were quantified by calculating interaction energies, which accounted for molecular conformation, charge distribution, bond angles, and hydrogen bonding contributions. The resulting poses were ranked by their interaction energies, and top-performing conformations were filtered to pinpoint drug candidates with the highest potential for effective binding to FOLH1.

### 4.6. Clustering analysis of drug molecules using K-means

To investigate the structural and activity-related patterns among drug molecules matched to ligands, we applied the K-means clustering algorithm to a combined dataset of Morgan fingerprints and standardized pIC_50_ values. Groups data points into clusters based on feature similarity, enabling the identification of molecular subsets with shared structural and functional properties. The optimal number of clusters was determined using two established techniques: Elbow Method [52] and the Silhouette Score Method [53]. We calculated the Sum of Squared Errors (SSE) for a range of cluster numbers. For each candidate cluster number, the Silhouette Score was computed to assess cluster quality. The pIC_50_ values were standardized (mean = 0, standard deviation = 1) to normalize their scale relative to the Morgan fingerprint features, ensuring equitable contribution to the clustering outcome. PCA projects the data into a lower-dimensional space by maximizing variance retention, providing a view of the drug compounds’ structural and activity-based characteristics.

#### 4.6.1. Structure-Activity Relationship (SAR) and Maximum Common Substructure (MCS) analysis.

We conducted a SAR [54] analysis using Seaborn’s JointGrid module to generate joint distribution plots (Joint Plots). For each cluster, joint plots were created to depict the distribution of pIC_50_ values against key molecular descriptors, such as molecular weight or hydrophobicity (LogP). Within each cluster, we calculated the Pearson correlation coefficient between the number of hydrogen bond acceptors and pIC_50_ values. To further characterize the drug candidates, we categorized their biological activity data (pIC_50_) and identified conserved structural motifs within clusters. The pIC_50_ values of the candidate drugs were classified into discrete activity levels (e.g., low, medium, high) based on predefined thresholds. Using the rdFMCS.

## 5. Experimental validation

### 5.1 Cell culture

LNCaP cells (MeisenCTCC, CTCC-400–0229, China) were maintained in specialized cell culture medium (Procell, CM-0143, China) at 37°C with 5% CO₂. Medium was replaced every 2–3 days, and cells were passaged at 80% confluence. Log-phase cells were used for subsequent experiments.

### 5.2 Cell proliferation assay

Cells (2,000/well) were seeded in 96-well plates and incubated for 24 hours. Melatonin (MedChemExpress, HY-B0075) was added at varying concentrations. After 24 hours, CCK-8 reagent (Solarbio, CA1210, China) was applied, and absorbance at 450 nm was measured using a microplate reader. Data were analyzed with Excel (Microsoft Office LTSC 2021) and GraphPad Prism (v10.0.2).

### 5.3. Confocal imaging

Log-phase LNCaP cells were seeded on poly-L-lysine (0.1 mg/mL, Procell, PB180523, China)-coated confocal dishes (NEST, 801001, China). After 24 hours, PBS (Servicebio, G4202-500ml, China) was added to the control group, while melatonin (0, 0.25, 0.5, or 1.0 mM) was added to the experimental group. After 24 hours, fixation with 4% paraformaldehyde (Solarbio, P1110, China), cells were stained with Anti-FOLH1 antibody (abcam, ab76104, USA) and Goat Anti-Rabbit IgG H&L (Alexa Fluor 488, ab150077, USA), then imaged using a Leica TCS-SP8 (Leica) confocal microscope.

### 5.4. Flow cytometry

Cells were divided into treatment and control groups. The treatment group received melatonin, while the control group received an equivalent volume of PBS, and after 24 hours, the cells were labeled with PE anti-human CD81 (Biolegend, 349505, USA) and APC anti-human FOLH1 (Biolegend, 342507. USA). Isotype controls included PE anti-human CD81 (Biolegend, 349505, USA) and APC Mouse IgG1, κ Isotype Ctrl (Biolegend, 400121, USA). Mean fluorescence intensity (MFI) of FOLH1 was analyzed using a BD Calibur2 flow cytometer.

### 5.5. Transwell migration and invasion assays

For invasion assays, matrix gel (Beyotime, C0383, China) diluted 1:8 in serum-free medium was added to Transwell chambers (Corning, 3422, USA) and incubated at 37°C for 3 hours. Lower chambers contained 600 µL of 20% FBS medium, while upper chambers received 100 µL of serum-free cell suspension. After 24 hours, cells were fixed with 4% paraformaldehyde, stained with crystal violet, and counted using ImageJ. The Transwell migration assay is similar to the invasion assay, except that the step of spreading the basement membrane gel is omitted. The rest of the methods are the same.

As for the control group, only PBS was added.

### 5.6. In Vivo Xenograft model and serum melatonin measurement

We randomly assigned nude mice to different experimental groups using a random number table. Nine 5-week-old male nude mice (purchased from the Department of Animal Science of Peking University Health Science Center) were divided into three groups under controlled lighting: normal circadian rhythm (12 h–12 h light–dark, lights on at 06:00 daily), constant darkness (12 h–12 h dark–dark), and disrupted rhythm (random light/dark cycles, placing the animals in altered light-cycle conditions with an 8-h light advance every 2–3 days), with food and water ad libitum When multiple time points were investigated simultaneously, light-tight cabinets were used to shift animals to the respective phase prior to the experiments. Treatment times correspond to Zeitgeber time (ZT) and indicate the timing relative to lights on in the animal facility such that ZT4 is 4 h after lights on, ZT10 is 10 h after lights on, ZT16 is 4 h after lights off and ZT22 is 10 h after lights off. LNCaP cells (5 × 10⁵ cells in 100 µL PBS&matrix gel (Beyotime, C0383, China) were subcutaneously injected. Tumor volume was monitored, and mice were sacrificed on day 20. We dissected the mice to obtain tumors. The tumors were then embedded in paraffin and sectioned. Following this, a primary antibody (Servicebio, GB115710–100, China) was added, along with a secondary antibody (Servicebio, GB25303, China) and DAPI (Servicebio, G1012, China). Finally, the samples were mounted (Servicebio, G1401, China) and immunofluorescence scanning was performed with 3DHISTECH (Pannoramic MIDI, Hungary). Serum melatonin levels were measured at day 18 using an ELISA kit (Meimian, KT2227-B, China). Animal studies were approved by the Peking University Institutional Animal Care Committee (DLASBE0137).

### 5.7. Hematoxylin and eosin (H&E) staining

The tumor from mice were fixed in 4% paraformaldehyde for 24 hours. Following fixation, the samples were subjected to paraffin embedding and subsequently sectioned into 4-μm slices. For histopathological analysis, the sections were stained using the hematoxylin-eosin staining (Servicebio, G1004, China) according to the standard protocol. The stained sections were then imaged using an optical microscope (E100, Nikon Corporation, Japan).

### 5.8. Immunohistochemistry

Paraffin sections were dewaxed using an environmentally friendly dewaxing solution and subsequently washed with 100% ethanol (Servicebio, 100092683, China) and distilled water. The sections were then treated with a 10 mM citric acid antigen repair solution (Servicebio, G1202, China) at high power in a microwave oven for 3 minutes to reach the boiling point, followed by treatment at low power for 15 minutes. The sections were allowed to cool naturally to room temperature. After cooling, the sections were washed with PBS solution and then blocked at room temperature for 1 hour using Tris-buffered saline containing 3% bovine serum albumin (Servicebio, GC305010, China) (Servicebio, G1206, China). Following blocking, the samples were incubated with the primary antibody (Servicebio, GB115710, China) at 4 °C overnight. The sections were then washed three times with PBS solution. Subsequently, the samples were incubated with the secondary antibody (Servicebio, GB23303, China) at room temperature for 50 minutes. The treated sections were washed again three times with PBS solution, followed by the addition of DAB chromogenic solution (Servicebio, G1212, China) and hematoxylin. After dehydration and sealing, the sections were prepared for imaging.

## 4. Statistical analysis of experiment

Data are presented as mean ± SD. GraphPad Prism (v10.0.2) was used for statistical analysis. Student’s t-test or ANOVA was used for functional analysis. When significant differences among multiple groups were detected by ANOVA, Tukey’s HSD post-hoc test was used to perform pairwise comparisons to identify which specific groups differed significantly. In cases where data did not meet the assumptions of normality, non-parametric tests were used. Specifically, the Kruskal-Wallis test was applied for multiple group comparisons, followed by Dunn’s post-hoc test for pairwise comparisons. *P* < 0.05 was considered statistically significant (*p < 0.05, **p < 0.01, ***p < 0.001).

## 5. Results

**Fig 1:**
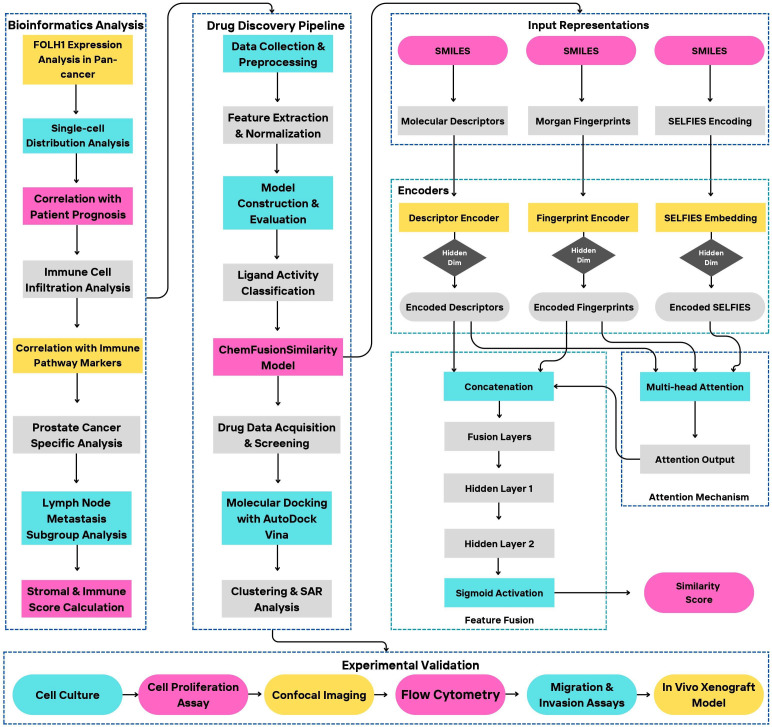
Comprehensive Research Framework for FOLH1-targeted Prostate Cancer Drug Discovery and Validation. Schematic representation of the three-phase research workflow integrating bioinformatics analysis, computational drug discovery, and experimental validation. The bioinformatics phase encompasses FOLH1 expression analysis across cancer types, single-cell distribution, correlation with patient outcomes, immune infiltration analysis, and prostate cancer-specific characterization. The drug discovery phase employs machine learning approaches, including the novel ChemFusionSimilarity model, for identifying potential FOLH1-targeting drugs, followed by molecular docking and structure-activity relationship analysis. The experimental validation phase confirms findings through in vitro and in vivo studies, including cell proliferation, imaging, flow cytometry, migration/invasion assays, and xenograft models.

Our study followed a systematic multi-phase approach to identify and validate potential therapeutic drugs targeting FOLH1 in prostate cancer (Fig 1). The research framework consisted of three interconnected phases: bioinformatics analysis, computational drug discovery, and experimental validation. In the first phase, we conducted bioinformatics analyses to characterize FOLH1 expression patterns and their clinical implications. The bioinformatics findings informed our computational drug discovery phase, where we developed a machine learning pipeline to identify potential FOLH1-targeting drugs. This systematic approach led to the identification of several promising drug candidates including melatonin. The subsequent experimental validation phase confirmed our computational predictions, demonstrating melatonin’s significant inhibitory effects on FOLH1 expression and prostate cancer cell functions, which will be detailed in the following sections.

## 6. Bioinformatic analysis

### 6.1. Pan-cancer expression profile of FOLH1

**Fig 2:**
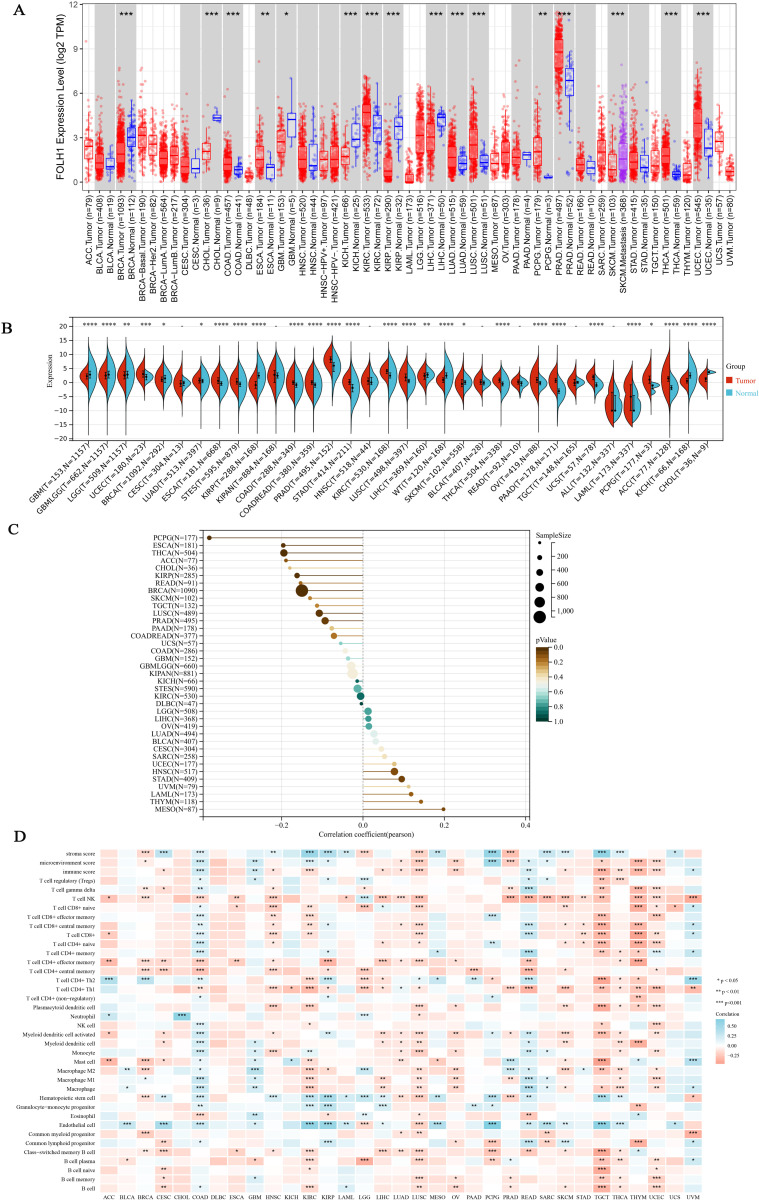
FOLH1 mRNA Expression Profiles in Human Normal and Tumor Tissues, and Immune Infiltration Patterns. **(A)** Boxplots demonstrating FOLH1 mRNA expression levels in normal vs. tumor tissues using TCGA database data. Tumor tissues are denoted by red dots and boxes, while normal tissues are represented by blue dots and boxes. **(B)** Violin plots illustrating FOLH1 mRNA expression in normal and tumor tissues using combined TCGA+GTEx database data. Tumor tissues are shown in orange boxes and normal tissues in blue boxes. **(C)** Pan-cancer analysis of Pearson correlation between FOLH1‌‌ expression and age (lollipop plot). **(D)** Correlation heatmap between FOLH1 expression and immune cell infiltration across cancers (XCELL algorithm-based). Symbols ns, *, **, and *** denote non-significant, P < 0.05, P < 0.01, and P < 0.001, respectively.

To investigate the expression profile of FOLH1 in tumor versus normal tissues, we initially compared FOLH1 mRNA levels between cancerous and adjacent normal tissues using TCGA database. Analysis revealed significant differences in FOLH1 expression between tumor and normal tissues across 15 cancer types, as well as differential expression between primary tumors and metastatic lesions in SKCM. Notably, FOLH1 exhibited predominant overexpression in tumor tissues across most evaluated cancers, with exceptions observed in BRCA, CHOL, GBM, KICH, KIRP, and LIHC (Fig 2A). Due to limited or absent normal tissue samples for certain cancers in TCGA, we performed expanded analyses integrating TCGA and GTEx datasets. This yielded expression data for 34 cancer types, demonstrating significant FOLH1 upregulation in 17 malignancies and downregulation in 10 (Fig 2B). Through single-cell distribution analysis of FOLH1 expression patterns across various tissues, it has been demonstrated that the primary distribution of FOLH1 shows tumor-type specificity, with predominant localization observed in glandular epithelial cells, squamous epithelial cells, specialized epithelial cells, endothelial cells, and mesenchymal cells, respectively (S1A Fig). Striking upregulation was observed in prostate cancer, underscoring the potential of FOLH1 as a tumor biomarker.

In different stratifications, the expression pattern of FOLH1 was analyzed in relation to patient characteristics. The results indicated that FOLH1 was associated with multiple relevant factors of different tumors. Specifically, in prostate cancer, it was observed that FOLH1 expression was higher in cases with lymph node metastasis (S1B Fig), and a significant negative correlation was presented in the age stratification (Fig 2C). In other stratification scenarios, although there was no statistical difference in FOLH1 expression, a significant high expression was observed in all cases.

### 6.2. FOLH1 expression and immune cell infiltration

The tumor microenvironment (TME) is a complex milieu supporting tumor cell survival, composed predominantly of immune cells, stromal components, and associated intra- and extracellular molecules. Tumor immune cell infiltration represents a critical component of neoplastic ecosystems, closely linked to tumorigenesis, progression, and metastasis. While prior studies have established associations between FOLH1 expression and prognosis, its correlation with immune infiltration remains underexplored. Utilizing multiple immune prediction algorithms, we analyzed the relationship between FOLH1 expression and immune infiltration levels. Heatmap analysis revealed robust associations between FOLH1 and diverse immune cell populations across pan-cancer datasets. Specifically, FOLH1 exhibited positive correlations with immune infiltration in BRCA, HNSC, LUSC, LUAD, LIHC, TGCT, THCA, THYM, UCEC and PRAD. Conversely, negative correlations were observed in COAD, GBM, KIPR, READ and UVM (Figs 2D, S2A-S2E). Leveraging the UCSC database, we identified significant associations between immune-modulating genes and FOLH1 expression across most tumor types (S3 Fig). FOLH1 expression demonstrated widespread positive correlations with both immunosuppressive and immunostimulatory genes. However, in specific cancers including TGCT, LUSC, HNSC, ESCA and NB, FOLH1 displayed negative correlations with MHC molecule-encoding genes. In TGCT, FOLH1 inversely correlated with immunosuppressive genes, while in LUSC and GBM, it showed negative associations with immunostimulatory genes.

### 6.3. FOLH1 in prostate cancer: expression, single-cell and immune infiltration analysis

**Fig 3:**
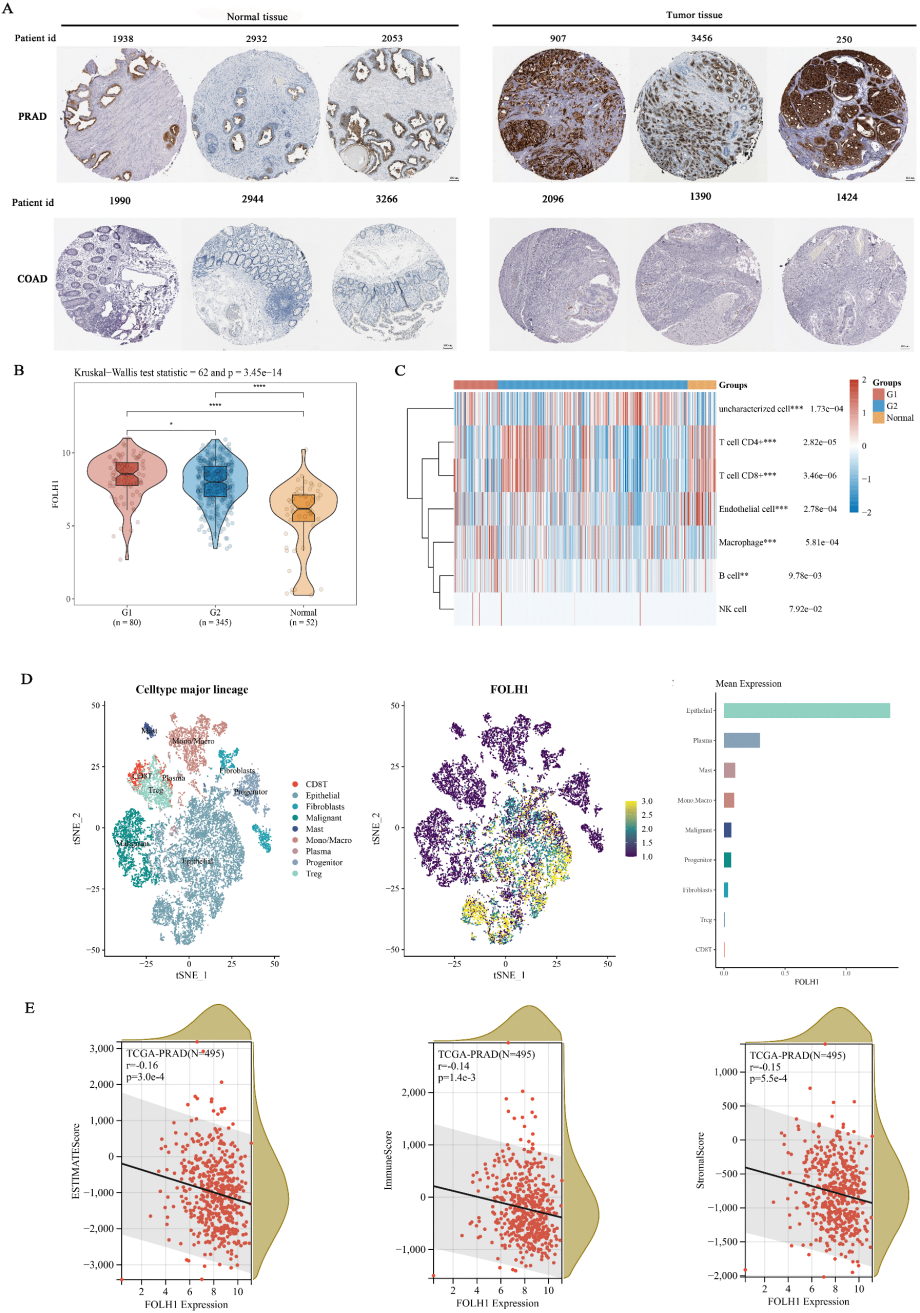
FOLH1 expression patterns in prostate and colon tissues. **(A)** Representative immunohistochemical staining images of FOLH1 in normal prostate tissue vs. prostate tumor tissue, and normal colon tissue vs. colon tumor tissue, these comparisons illustrate the differential expression and diagnostic potential of FOLH1 across different malignancy types. **(B)** Composite visualization of FOLH1 expression distribution in tumor vs. normal tissues through violin plots, dot plots, and boxplots (significance determined by Kruskal-Wallis test comparing G2 [lymph node metastasis-negative group]; G1 [lymph node metastasis-positive group]). **(C)** Percentage abundance of tumor-infiltrating immune cells per sample (stratified by lymph node metastasis status), with significance assessed via Kruskal-Wallis test. Asterisks denote significance levels: *p < 0.05, **p < 0.01, ***p < 0.001. **(D)** Single-cell clustering analysis: Left panel - UMAP plot showing FOLH1 expression distribution across cell types (color-coded by cell type); Color gradient represents expression intensity (darker hues = lower expression, brighter hues = higher expression). Right panel - Bar plot illustrating FOLH1 expression abundance in different cell populations. **(E)** Pearson’s correlation coefficients between FOLH1 expression and three immune infiltration ‌‌scores in prostate adenocarcinoma (PRAD).

Given the significantly elevated expression of FOLH1 in prostate cancer and its association with multiple clinicopathological factors, we focused on prostate cancer to investigate FOLH1 expression patterns. Immunohistochemical staining demonstrated markedly higher FOLH1 expression in prostate cancer tumor tissues compared to normal counterparts (Fig 3A). Further analysis revealed differential FOLH1 expression between the G2 cohort (non-lymph node metastasis group) and G1 cohort (lymph node metastasis group), with both groups showing significantly elevated expression relative to normal controls (Fig 3B). Immune infiltration analysis demonstrated correlations between lymph node metastasis status and infiltration levels of B cells, macrophages, and mast cells (Fig 3C). UMAP visualization revealed that FOLH1 predominantly clustered within epithelial cells, which constituted the major cellular component (Fig 3D). FOLH1 expression exhibited negative correlations with all three immune infiltration scoring metrics (stromal, immune, and ESTIMATE scores) in PRAD (Fig 3E). Prognostic analysis indicated that elevated FOLH1 expression may serve as an adverse prognostic factor in PRAD.

## 7. Computational drug discovery

### 7.1. Identification of target proteins and ligand dataset construction

The number of FOLH1 ligands with IC_50_ values was limited (n = 420), this dataset alone was insufficient to support reliable pIC_50_ model training. We identified several prostate cancer-related proteins strongly associated with FOLH1 based on ProteinAtlas evidence scores, including PBOV1 (Prostate and Breast Cancer Overexpressed 1), KLK3 (Kallikrein-Related Peptidase 3), CTAG2 (Cancer/Testis Antigen 2), PSCA (Prostate Stem Cell Antigen), ESR1 (Estrogen Receptor 1), and NKX3–1 (NK3 Homeobox 1). Ligand activity data for these correlated targets were then integrated from multiple chemical databases, yielding an expanded training set of 28,241 ligands for pIC_50_ prediction.

### 7.2. Performance of machine learning models

**Fig 4:**
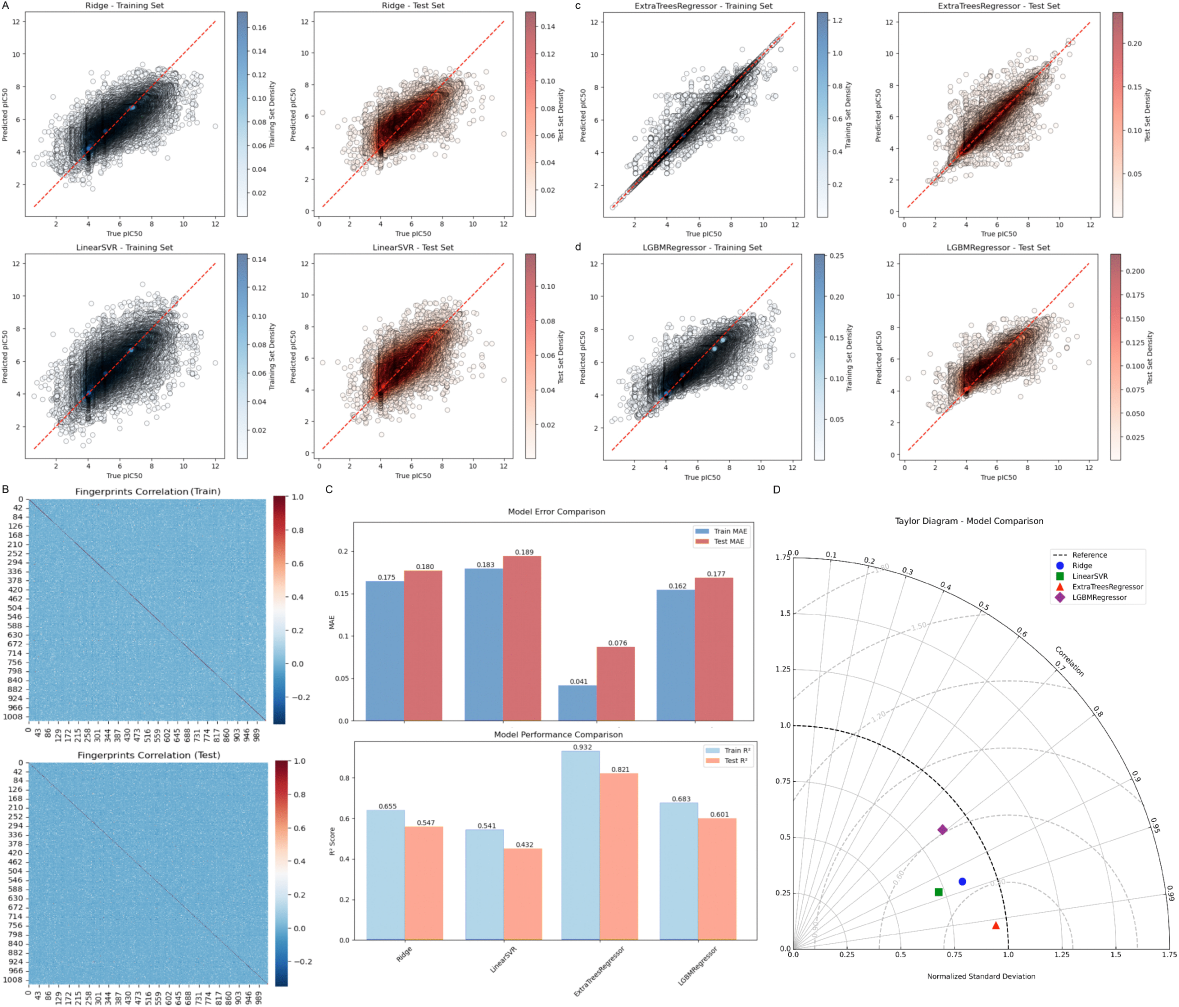
Comparative Analysis of Machine Learning Models for pIC_50_ Prediction. Performance evaluation of machine learning models for predicting ligand activity (pIC_50_). **(A)** Scatter plots with Gaussian kernel density estimation showing the correlation between experimental and predicted pIC_50_ values for four machine learning models: Extra Trees Regressor, Linear SVR, Ridge Regression, and LightGBM Regressor, diagonal line represents perfect prediction. **(B)** Correlation heatmap of Morgan fingerprints (1024 bits) across training and test sets, with high self-correlation (red) indicating consistency within molecular representations and low inter-fingerprint correlation demonstrating the model’s ability to distinguish unique molecular features. **(C)** Quantitative performance metrics including Mean Absolute Error (MAE) and coefficient of determination (R²) for both training and test sets across all models. **(D)** Taylor diagram illustrating the comparative performance based on correlation with experimental data, standard deviation, and root-mean-square error (RMSE), closest to the reference point (correlation = 1.0) represents superior predictive accuracy.

To evaluate the predictive accuracy of ligand activity, expressed as pIC_50_ values, we compared eight machine learning models: Ridge Regression (Fig 4Aa), Linear SVR (Fig 4Ab), Extra Trees Regressor (Fig 4Ac), LightGBM Regressor (Fig 4Ad), XGBoost, Random Forest, CNN, and GNN (S4A Fig). The Extra Trees Regressor exhibited the strongest alignment with the regression line, with a high density of points concentrated along the diagonal, indicating superior predictive accuracy and consistency. In contrast, Linear SVR displayed a widely dispersed pattern with sparse density, suggesting poor predictive capability. Ridge Regression showed moderate dispersion with a notable number of outliers, while LightGBM Regressor produced an overly clustered distribution near the regression line, albeit with scattered outliers. These observations collectively suggest that the Extra Trees Regressor outperformed the other models in capturing the relationship between experimental and predicted pIC50 values. To further dissect the Extra Trees Regressor’s performance, we examined the correlation of Morgan fingerprints (1024 bits) across the training and test sets (Fig 4B). A correlation heatmap revealed that each fingerprint bit exhibited high self-correlation (visualized in red), indicating strong consistency within individual molecular representations.

Model performance was quantified using two key metrics: Mean Absolute Error (MAE) and the coefficient of determination (R²). Extra Trees Regressor achieved the lowest MAE values (training set: 0.041, test set: 0.076) and the highest R² scores (training set: 0.932, test set: 0.821), demonstrating exceptional predictive accuracy and model fit across both datasets. Linear SVR recorded the highest MAE (training set: 0.183, test set: 0.189) and lowest R² (training set: 0.541, test set: 0.432), indicating the weakest performance among the models. Ridge Regression produced moderate MAE values (training set: 0.175, test set: 0.180) and R² scores (training set: 0.655, test set: 0.547), reflecting intermediate predictive capability. LightGBM Regressor showed competitive MAE (training set: 0.162, test set: 0.177) and R² (training set: 0.683, test set: 0.601), but its performance was slightly inferior to that of Extra Trees Regressor (Fig 4C). These metrics confirm that the Extra Trees Regressor consistently outperformed the other models, achieving the best tradeoff between accuracy and generalization. To facilitate candidate prioritization, we additionally evaluated model performance on a secondary binary classification task, in which pIC_50_ values were discretized into high-activity and low-activity classes (Section 2.3.2 for thresholds). Further classification metrics revealed the Extra Trees Regressor’s superior performance, with cross-validation results showing accuracy of 0.9452, precision of 0.9374, recall of 0.9429, and F1-score of 0.9351, while test results demonstrated accuracy of 0.9126, precision of 0.9074, recall of 0.9206, and F1-score of 0.9141 (Table 1).

**Table 1:** Comparison of Cross-Validation and Test Set Performance Metrics for Machine Learning Models in Effective Drug Compound Activity Prediction.

	Cross-Validation Results				Test Results			
Accuracy^a^	Precision^b^	Recall^c^	F1-Score^d^	Accuracy^e^	Precision^f^	Recall^g^	F1-Score^h^
Ridge^i^	0.9217	0.9095	0.9012	0.9038	0.8048	0.8387	0.7667	0.7908
LinearSVR^j^	0.7471	0.7596	0.7624	0.7568	0.7714	0.7832	0.7619	0.7699
ExtraTrees^k^	0.9452	0.9374	0.9429	0.9351	0.9126	0.9074	0.9206	0.9141
LGBMRegressor^l^	0.8905	0.9059	0.8714	0.8883	0.7861	0.7832	0.7124	0.7161
XGBoost^m^	0.9429	0.9209	0.9048	0.9382	0.7381	0.7687	0.7381	0.7388
RandomForest^n^	0.9262	0.9382	0.9138	0.9269	0.7595	0.7887	0.7524	0.7602
CNN^o^	0.8476	0.8286	0.8125	0.8354	0.7048	0.7278	0.7063	0.7029
GNN^p^	0.7269	0.7667	0.7586	0.7206	0.6381	0.6333	0.6386	0.6359

The performance of various machine learning models evaluated for drug screening. The classification metrics (accuracy, precision, recall, and F1-score) reported in this table were obtained from a secondary binary classification task, in which predicted pIC_50_ values were discretized into high-activity (pIC_50_ > 7) and low-activity (pIC_50_ ≤ 7) classes. These metrics should not be conflated with the primary regression task (MAE, R^2^) reported in Fig 4C.The metrics include ^a^Accuracy, overall correctness of predictions on the cross-validation sets; ^b^Precision, proportion of correctly predicted high-activity class out of all predicted high-activity class on the cross-validation sets; ^c^Recall, proportion of correctly predicted high-activity classout of all actual high-activity classon the cross-validation sets; ^d^F1-Score, the harmonic mean of precision and recall on the cross-validation sets. The Cross-Validation Results were obtained through a 5-fold cross-validation procedure, providing a robust estimate of the model’s generalization ability. The metrics on the independent test set are ^e^Accuracy, overall correctness of predictions on the test set; ^f^Precision, proportion of correctly predicted high-activity class out of all predicted high-activity class_0_ on the test set; ^g^Recall, proportion of correctly predicted high-activity class_0_ out of all actual high-activity class on the test set; and ^h^F1-Score, the harmonic mean of precision and recall on the test set. The models evaluated are ^i^Ridge, Ridge Regression; ^j^LinearSVR, Linear Support Vector Regression; ^k^ExtraTrees, Extra Trees Regressor; ^l^LGBMRegressor, Light Gradient Boosting Machine Regressor; ^m^XGBoost, Extreme Gradient Boosting; ^n^RandomForest, Random Forest; ^o^CNN, Convolutional Neural Network; and ^p^GNN, Graph Neural Network.

A Taylor diagram was employed to visualize the comparative performance of the four models based on their standard deviation, correlation with experimental data, and root-mean-square error (RMSE). The Extra Trees Regressor was positioned closest to the reference point (correlation = 1.0, standard deviation normalized to 0.3), signifying its superior alignment with experimental pIC_50_ values and minimal predictive error. LightGBM Regressor and Ridge Regression also approached the 1.0 correlation axis but were located at greater distances (LightGBM beyond 0.6, Ridge Regression between 0.3 and 0.6), indicating higher variance and error. Linear SVR, positioned between 0.3 and 0.6 but far from the 1.0 correlation line (Fig 4D), underscored its poor predictive reliability.

Perturbation analysis was conducted to assess the robustness of each model to controlled input perturbations (S4A Fig). The results revealed significant differences in model stability. Most models, including Ridge, LinearSVR, RandomForest, LGBMRegressor, XGBoost, SVM, and, where applicable, CNN and GNN, exhibited relatively large fluctuations in MAE and R^2^ when subjected to perturbations. These fluctuations suggest sensitivity to input variations, which could impact their reliability in real-world scenarios with noisy or incomplete data. In contrast, the Extra Trees Regressor demonstrated remarkable stability. Its MAE and R² metrics showed negligible fluctuations under perturbation, with MAE rising only gradually and R² declining slowly as perturbation intensity increased. The final hyperparameter configurations for all machine learning models used in this study are detailed in Table 2, which includes both tuned parameters, optimized via grid search during 5-fold cross-validation, and default parameters.

**Table 2:** Final Hyperparameter Configurations for Machine Learning Models in Drug Compound Activity Prediction.

Model	Hyperparameter	Value	Tuned or Default	Notes
Ridge^a^	alpha	1.0	Tuned	Regularization strength, tuned via grid search
	fit_intercept	True	Default	Default setting in scikit-learn (v1.5.2)
	max_iter	1000	Default	Maximum iterations for convergence
LinearSVR^b^	C	0.1	Tuned	Regularization parameter, tuned via grid search
	epsilon	0.01	Tuned	Epsilon-tube for loss function, tuned via grid search.
	fit_intercept	True	Default	Default setting in scikit-learn (v1.5.2)
	max_iter	1000	Default	Maximum iterations for optimization
ExtraTrees^c^	n_estimators	200	Tuned	Number of trees, tuned via grid search
	max_depth	None	Default	No maximum depth restriction (full tree growth)
	min_samples_split	2	Default	Minimum samples required to split a node
	min_samples_leaf	1	Default	Minimum samples required at a leaf node
	max_features	auto	Tuned	Number of features to consider for splits (sqrt of total features)
LGBM Regressor^d^	learning_rate	0.05	Tuned	Learning rate, tuned via grid search
	n_estimators	300	Tuned	Number of boosting iterations, tuned via grid search
	max_depth	7	Tuned	Maximum tree depth, tuned via grid search
	num_leaves	31	Tuned	Maximum number of leaves per tree, tuned via grid search
	min_child_samples	20	Default	Minimum number of samples in a leaf (LightGBM v4.5.0)
	reg_lambda	0.0	Default	L2 regularization term on weights
XGBoost^e^	learning_rate	0.1	Tuned	Learning rate (eta), tuned via grid search
	n_estimators	200	Tuned	Number of boosting iterations, tuned via grid search
	max_depth	6	Tuned	Maximum tree depth, tuned via grid search
	lambda	1.0	Default	L2 regularization term on weights (XGBoost v3.0.1)
	alpha	0.0	Default	L1 regularization term on weights
	subsample	0.8	Tuned	Fraction of samples used per tree, tuned via grid search
GNN^f^	num_layers	3	Tuned	Number of GNN layers, tuned via grid search.
	hidden_dim	128	Tuned	Dimension of hidden node features, tuned via grid search
	aggregation	mean	Tuned	Aggregation function for message passing
	learning_rate	0.001	Tuned	Learning rate for Adam optimizer, tuned via grid search
	dropout	0.2	Tuned	Dropout rate for regularization, tuned via grid search
	batch_size	64	Tuned	Batch size for training, tuned via grid search
CNN^g^	num_conv_layers	2	Tuned	Number of convolutional layers, tuned via grid search
	filters	[64, 128]	Tuned	Number of filters per layer, tuned via grid search
	kernel_size	3	Tuned	Size of convolutional kernels, tuned via grid search
	pooling	max	Tuned	Pooling strategy (max pooling)
	learning_rate	0.001	Tuned	Learning rate for Adam optimizer, tuned via grid search
	dropout	0.3	Tuned	Dropout rate for dense layers, tuned via grid search
	batch_size	32	Tuned	Batch size for training, tuned via grid search
Random Forests^h^	n_estimators	150	Tuned	Number of trees, tuned via grid search
	max_depth	None	Default	No maximum depth restriction (full tree growth)
	min_samples_split	2	Default	Minimum samples required to split a node
	min_samples_leaf	1	Default	Minimum samples required at a leaf node
	max_features	auto	Tuned	Number of features to consider for splits (sqrt of total features)

The final hyperparameters used for Each model in the reported results, including both tuned parameters, optimized via grid search during 5-fold cross-validation, and default parameters. Models were implemented using standard machine learning libraries, with specific versions noted where applicable. All models were trained on the feature matrix derived from the training set.The models evaluated are ^a^Ridge, Ridge Regression; ^b^LinearSVR, Linear Support Vector Regression; ^c^ExtraTrees, Extra Trees Regressor; ^d^LGBMRegressor, Light Gradient Boosting Machine Regressor; ^e^XGBoost, Extreme Gradient Boosting; ^f^GNN, Graph Neural Network; ^g^CNN, Convolutional Neural Network; and ^h^RandomForest, Random Forest.

### 7.3. Clustering and dimensionality reduction of FOLH1 Ligand Dataset

**Fig 5:**
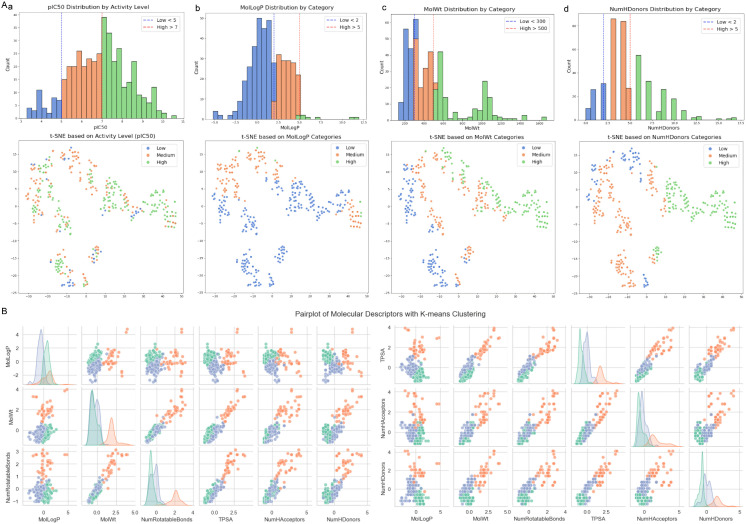
Molecular Descriptor Analysis and Clustering of FOLH1 Ligands. (A) t-SNE visualization of ligand distribution colored by: (a) activity categories (low: pIC_50_ < 5; medium: 5 ≤ pIC_50_ ≤ 7; high: pIC_50_ > 7), showing high-activity ligands with broad structural diversity, medium-activity ligands forming compact clusters, and sparse distribution of low-activity ligands; **(b)** LogP values (< 2, 2-5, > 5), revealing dense clustering for LogP < 5 compounds and dispersion for the rare LogP > 5 compounds; (c) molecular weight (< 500 Da, > 500 Da), demonstrating tight clustering of predominant lower-weight compounds and sparse distribution of higher-weight molecules; (d) number of hydrogen bond donors (< 2, 2-5, > 5), showing dense clusters for compounds with fewer donors and sparse distribution for those with > 5 donors. **(B)** Pairwise relationship plots of key molecular descriptors (MolLogP, MolWt, NumRotatableBonds, TPSA, NumHAcceptors, NumHDonors) colored by K-means clusters, illustrating three distinct clusters with separation based on physicochemical properties.

To elucidate the structural and physicochemical diversity within the FOLH1 ligand dataset (n = 420, detailed in S1 File), we integrated pIC_50_ values with molecular descriptors characterizing structure and properties. This dataset was subjected to K-means clustering, dimensionality reduction, and subsequent computational analysis. The ligands were classified into three activity categories based on pIC_50_ thresholds: low activity (pIC_50_ < 5), medium activity (5 ≤ pIC_50_ ≤ 7), and high activity (pIC_50_ > 7). Among these, high-activity ligands (pIC_50_ > 7) were the most abundant, while low-activity ligands (pIC_50_ < 5) were the least represented. Medium-activity ligands (5 ≤ pIC_50_ ≤ 7) fell between these extremes in quantity. A t-SNE plot (Fig 5Aa) was generated to visualize the distribution of these ligands in a two-dimensional space. High-activity ligands exhibited a broad spread, indicating substantial structural diversity. In contrast, medium-activity ligands formed a more compact cluster, suggesting greater structural similarity within this group. Low-activity ligands were sparsely distributed, consistent with their smaller population. The majority of ligands had LogP values below 5, with the highest frequency observed for LogP < 2 (Hydrophobicity, Fig 5Ab). Only six ligands exhibited LogP > 5. The t-SNE visualization revealed that ligands with LogP < 2 and LogP between 2 and 5 formed dense clusters, whereas those with LogP > 5 were markedly dispersed, reflecting their rarity and structural divergence. Most ligands had molecular weights below 500 Da, constituting the predominant group. In the t-SNE plot, these ligands appeared tightly clustered, indicating homogeneity (MolWt, Fig 5Ac). The number of hydrogen bond donors was predominantly below 5, with a notable subset below 2 (NumHDonors, Fig 5Ad). Ligands with more than 5 hydrogen bond donors were infrequent.

K-means clustering was applied to the standardized molecular descriptor data, resulting in the identification of three distinct clusters. These clusters were subsequently analyzed using pairwise relationship plots generated with Seaborn’s Pairplot functionality (Fig 5B). The plots encompassed key descriptors: MolLogP (hydrophobicity), MolWt (molecular weight), NumRotatableBonds (number of rotatable bonds), TPSA (topological polar surface area), NumHAcceptors (number of hydrogen bond acceptors), and NumHDonors (number of hydrogen bond donors). The pairwise visualizations delineated the three clusters, revealing their separation based on descriptor values. For instance, the MolLogP plot highlighted a cluster with predominantly low LogP values (< 2), while the MolWt plot distinguished a tightly grouped cluster of ligands below 500 Da from more scattered outliers. Similarly, NumHDonors and NumHAcceptors plots underscored the prevalence of ligands with fewer hydrogen-bonding groups, with distinct clustering patterns emerging across all descriptors.

### 7.4. Comparative analysis of ChemFusionSimilarity and Tanimoto similarity metrics

To assess the performance of the deep learning-based ChemFusionSimilarity model relative to the traditional Tanimoto similarity metric, we merged both similarity scores into a unified dataframe (S2 File). This dataset enabled a comprehensive comparison of the two approaches across multiple visualization techniques, providing insights into their distributional properties and predictive outcomes. A 3D density plot was constructed to visualize the relationship between ChemFusionSimilarity and Tanimoto similarity scores (S4B Fig). The ChemFusionSimilarity values predominantly ranged from 0.16 to 0.22, while Tanimoto similarity scores were largely concentrated between 0.10 and 0.14. Notably, the plot revealed no significant outliers or dispersion, indicating a consistent and compact distribution for both metrics. A joint distribution plot depicted the bivariate relationship between ChemFusionSimilarity and Tanimoto similarity, alongside their marginal distributions (S4C Fig). The joint plot demonstrated a strong linear correlation between the two metrics, with all data points aligning closely along the regression line and no observable outliers. The marginal distributions further confirmed the concentration of ChemFusionSimilarity scores (0.16–0.22) and Tanimoto scores (0.10–0.14), reinforcing the absence of discrete or aberrant values. This alignment highlights the consistency between the deep learning-based and traditional similarity measures, while also underscoring ChemFusionSimilarity’s tendency to assign higher similarity scores. Violin plots were employed to compare the distributional characteristics of ChemFusionSimilarity and Tanimoto similarity scores (S4D Fig). Compounds identified via ChemFusionSimilarity are consistently associated with higher pIC50 values than those retrieved using conventional structural similarity alone. Following the similarity analysis, the ChemFusionSimilarity model was used to predict IC_50_ values for the ligand-drug pairs. The resulting distribution of predicted IC_50_ values was visualized (S4E Fig), showing that the majority fell within the range of 300–400 nM, indicative of moderate to high potency. A smaller subset of predictions yielded IC_50_ values below 150 nM, representing ligands with exceptionally high activity.

We examined the relationship between Topological Polar Surface Area (TPSA) and drug compound activity (pIC_50_) as shown in S4F Fig. Compounds with TPSA values greater than 125 Å² consistently demonstrated lower activity, with pIC_50_ values predominantly falling between 5 and 6. In contrast, compounds with TPSA values below 125 Å² exhibited substantially higher activity, with pIC_50_ values typically ranging from 7 to 9. This pronounced threshold effect suggests that excessive polar surface area may hinder membrane permeability or target binding, highlighting TPSA as a crucial parameter for optimizing drug compound potency. To quantify the relative importance of various molecular descriptors in predicting compound activity, we conducted feature importance analysis (S4G Fig). The results identified Complementary Property Index (CPI) as the most influential descriptor with a coefficient magnitude of 3.24, indicating its substantial positive correlation with compound potency. In contrast, LogP exhibited the lowest impact with a coefficient magnitude of -0.41, suggesting a mild negative correlation between lipophilicity and activity within our dataset. Hydrogen bond donor count also demonstrated a negative correlation with activity, with a coefficient magnitude of -0.4, emphasizing the molecular properties that most significantly influence compound activity against the target of interest.

### 7.5. Drug candidate screening and similarity matching

**Fig 6:**
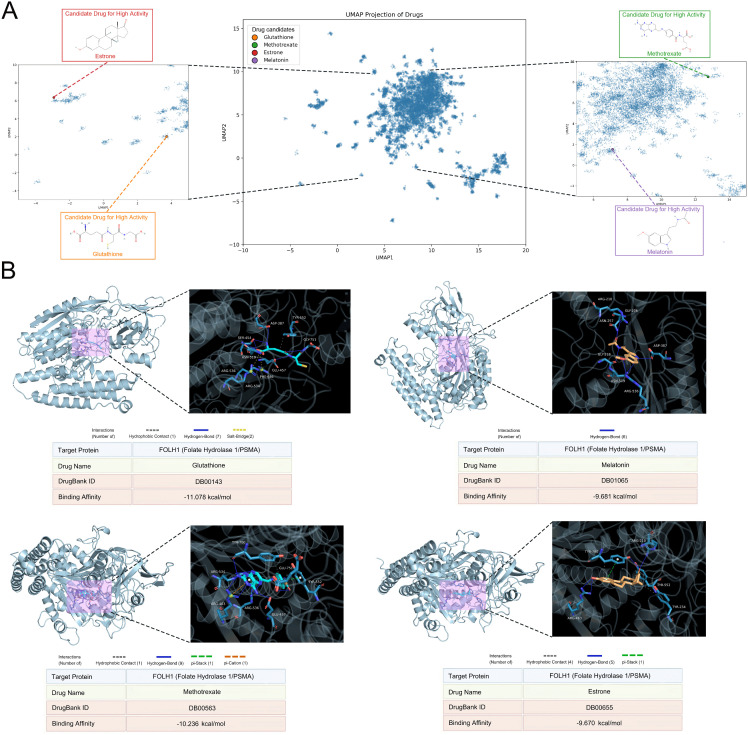
Top Drug Candidates and Molecular Docking Analysis with FOLH1. **(A)** Chemical structures of the four highest-scoring drug candidates: Glutathione, Methotrexate, Melatonin, and Estrone, selected based on their exceptional similarity scores with known FOLH1 ligands. **(B)** Molecular docking results visualized using Discovery Studio Visualizer and UCSF Chimera, showing binding poses and interaction networks for each candidate with FOLH1. The visualization highlights the spatial orientation of each ligand within the FOLH1 binding pocket and specific residues involved in key intermolecular interactions.

12,457 drug compounds from the DrugBank database was refined to 1,819 candidate drugs (S3 File). Among the 1,819 candidates, 36 exhibited similarity scores exceeding 0.8 with FOLH1 ligands (S4 File), indicating strong structural resemblance. From this subset, four drugs with the highest similarity scores were prioritized for detailed investigation: Glutathione, Methotrexate, Melatonin, and Estrone (Fig 6A). The docking results provided quantitative binding affinities (expressed in kcal/mol) and detailed insights into the intermolecular interactions stabilizing each ligand-protein complex (Fig 6B). Glutathione (Binding Affinity: -11.078 kcal/mol) exhibited the lowest predicted binding energy among the tested candidates, indicating a favorable theoretical interaction. The complex featured one hydrophobic contact, seven hydrogen bonds, and two salt bridges. The predominance of hydrogen bonds and ionic salt bridges suggests a highly stable interaction profile, likely driven by polar and charged residues within the FOLH1 binding pocket. Methotrexate (Binding Affinity: -10.236 kcal/mol) also demonstrated a comparable predicted binding affinity. This complex included one hydrophobic contact, nine hydrogen bonds, one pi-stacking interaction, and one pi-cation interaction. The extensive hydrogen bonding network, complemented by aromatic and cationic interactions, underscores Methotrexate’s capacity for robust and multifaceted engagement with FOLH1. Melatonin (Binding Affinity: -9.681 kcal/mol), indicative of favorable binding. The docking revealed six hydrogen bonds as the primary stabilizing forces. Estrone (Binding Affinity: -9.670 kcal/mol), closely comparable to Melatonin. This complex exhibited four hydrophobic contacts, five hydrogen bonds, and one pi-stacking interaction. The balanced contribution of hydrophobic and hydrogen-bonding interactions, enhanced by aromatic stacking, indicates a versatile binding mechanism tailored to the FOLH1 active site. It is important to note that the differences in predicted binding affinities among these top four candidates (ranging from -9.670 to -11.078 kcal/mol) are relatively small and fall within the expected uncertainty margin of standard molecular docking algorithms (typically ±1–2 kcal/mol).

### 7.6. Clustering analysis of candidate drugs

**Fig 7:**
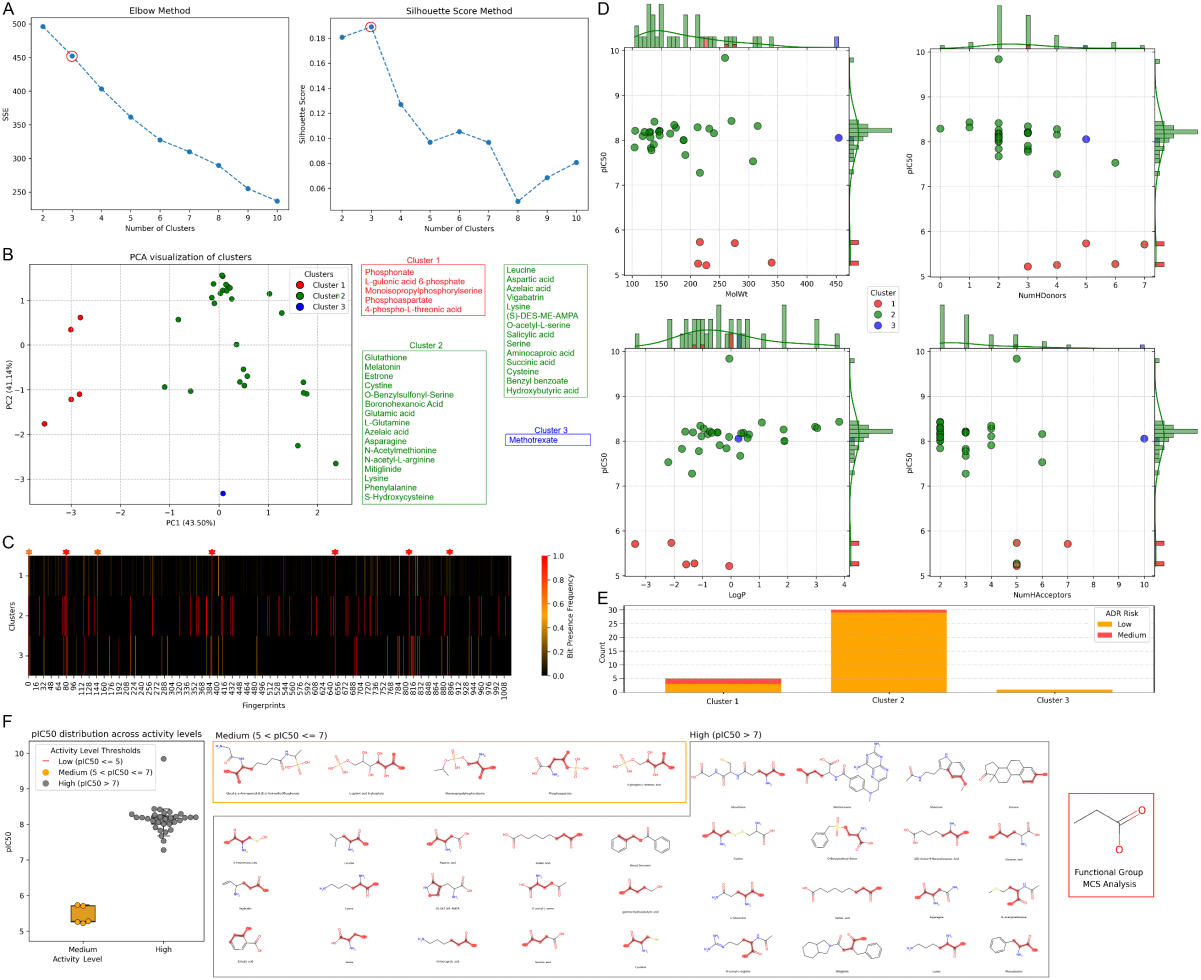
Clustering Analysis and Structural Characterization of FOLH1-Targeted Drug Candidates. **(A)** Cluster optimization using the Elbow Method (Sum of Squared Errors) and Silhouette Score Method. **(B)** PCA showing the distribution of the three clusters in two-dimensional space: Cluster 2 (upper-right quadrant) containing Glutathione, Melatonin, and Estrone; Cluster 1 (left region) encompassing the majority of candidates; and Cluster 3 (distinct position) consisting solely of Methotrexate. **(C)** Heatmap visualization of Morgan fingerprint bits (1024-bit representation) revealing cluster-specific structural patterns. **(D)** Physicochemical property analysis by cluster. **(E)** Adverse Drug Reaction risk analysis showing predominantly Low risk classification across all 36 candidates, with a small fraction categorized as Medium risk and none as High risk. **(F)** Maximum Common Substructure (MCS) analysis revealing a carbonyl group (C = O) as a common scaffold among moderately active drugs, while high-activity drugs consistently contained a hydroxyl group (OH), suggesting its importance for enhanced potency.

The optimal number of clusters was determined using two complementary methods, the Elbow Method and the Silhouette Score Method. The Elbow Method plotted the SSE against a range of cluster numbers, identifying an inflection point at three clusters with an SSE of 450, indicating a balance between clustering tightness and model complexity. The Silhouette Score Method corroborated this finding, with the highest score of 0.2 achieved for three clusters (Fig 7A). Principal Component Analysis (PCA) was employed to reduce the dimensionality of the clustered data into a two-dimensional space (Fig 7B). Cluster 2 was predominantly distributed in the upper-right quadrant and included three noteworthy drugs: Glutathione, Melatonin, and Estrone, suggesting shared structural or activity traits. Cluster 1 was concentrated in the left region, encompassing a broader set of candidates, while Cluster 3 consisted solely of Methotrexate, positioned distinctly from the others. This separation highlights Methotrexate’s unique molecular profile compared to the other clusters. A heatmap of Morgan fingerprint bits (1024-bit representation) revealed cluster-specific patterns (Fig 7C). Cluster 1 exhibited the highest frequency at bit 80, indicating a dominant structural motif. Cluster 2 showed frequent activation across multiple bits (80, 272, 416, 656, 880), suggesting greater structural diversity. Cluster 3, comprising only Methotrexate, was most frequent at bit 688. Common high-frequency bits across all clusters (80, 144, 384, 800, 896) indicate shared structural features, while cluster-specific patterns underscore their distinctiveness. Cluster 1 exhibited predominantly medium biological activity, with pIC_50_ values ranging from 0.5 to 0.7, and molecular weights (MolWt) between 200 and 350 Da. This cluster was further defined by a higher number of hydrogen bond donors, typically ranging from 3 to 7, and LogP values spanning -3–0, indicative of greater hydrophilicity. Additionally, the number of hydrogen bond acceptors varied from 5 to 7 (Fig 7D). Cluster 2 comprised compact, potent drugs with pIC50 values exceeding 7, indicating strong binding affinity (typically corresponding to IC50 values < 100 nM). The molecular weights of drugs in this cluster ranged from 200 to 350 Da, aligning well with the desirable range for oral bioavailability as per Lipinski’s Rule of Five. The number of hydrogen bond donors varied from 3 to 7, while hydrogen bond acceptors ranged from 5 to 7, suggesting a balanced capacity for polar interactions critical for target binding. LogP values spanned from -3–0, indicating moderate hydrophilicity, which is advantageous for solubility and membrane permeability in drug-like molecules. Notable drugs in Cluster 2 included Glutathione (307.08 Da, 6 HBD, 6 HBA, LogP -2.21), Melatonin (149.05 Da, 2 HBD, 3 HBA, LogP 0.15), and Estrone (270.16 Da, 1 HBD, 2 HBA, LogP 3.82). These molecules, despite their structural diversity, shared compact scaffolds and high potency, underscoring the cluster’s enrichment with drug-like candidates. Cluster 3, represented solely by Methotrexate, also displayed high activity (pIC_50_ > 7) but was distinguished by a significantly larger molecular weight of 450 Da. This cluster featured a moderate number of hydrogen bond donors (NumHDonors = 5) and a balanced LogP value of approximately 0, suggesting an equilibrium between hydrophilicity and hydrophobicity. With 10 hydrogen bond acceptors, the highest among the clusters, Cluster 3 highlighted Methotrexate as a larger, highly active compound with an extensive capacity for hydrogen-bond interactions (Fig 7D).

An Adverse Drug Reaction (ADR) risk analysis was conducted for all 36 candidates (Fig 7E). The majority of drugs were classified as Low risk, with only a small fraction categorized as medium risk. No candidates exhibited High risk, suggesting a generally favorable safety profile across the dataset. This assessment enhances the therapeutic potential of the identified drugs, particularly the high-activity candidates in Clusters 2 and 3. The MCS was computed for each cluster. The MCS featured a substructure with an oxygen atom doubly bonded to a carbonyl group, reflecting a common scaffold among moderately active drug compounds. High-activity drugs consistently included a hydroxyl group (OH) within their MCS, suggesting its role as a critical functional group for enhanced potency (Fig 7F).

## 8. Experimental validation

### 8.1. Melatonin suppresses FOLH1 expression and inhibits invasion and metastasis of LNCaP cells in vitro

**Fig 8:**
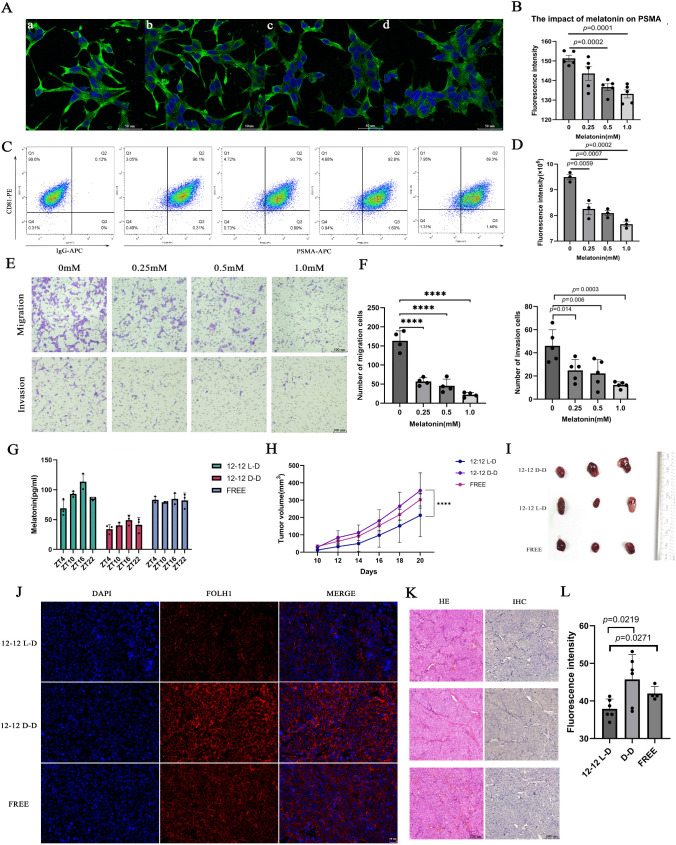
Melatonin suppresses FOLH1 expression and inhibits LNCaP metastasis/invasion. **(A)** Confocal images of LNCaP cells treated with different melatonin concentrations (0; 0.25; 0.5; 1.0 mM **(a-d)**), DAPI = blue, FOLH1 = green. **(B)** Statistical analysis of FOLH1 fluorescence intensity across concentrations (n = 5). C, **D.** Flow cytometric quantification of FOLH1 signal intensity (n = 3): **(C)** Left to right - isotype control, 0; 0.25; 0.5; 1.0 mM melatonin treatments. E, **F.** Invasion/migration assays: Control vs. melatonin-treated cells were seeded in Transwell upper chambers for 24H. Migrated cells were fixed with 4% paraformaldehyde, imaged, and counted from 5 random fields (bar plots). G-I. Circadian rhythm-dependent melatonin variations and corresponding tumor volumes. J, K, **L.** FOLH1 immunofluorescence staining, Pathological sections (HE staining), immunohistochemistry and intensity quantification in prostate cancer under different circadian conditions. Data presented as mean ± SD. *p < 0.05, **p < 0.01, ***p < 0.001.

To determine the optimal melatonin concentration for subsequent experiments, CCK-8 assays determined an IC₅₀ of 1.063 mM melatonin for LNCaP cells (S5A Fig). Based on these results, the maximum melatonin concentration applied in experiments was set at 1.0 mM. The effects of melatonin on FOLH1 expression were evaluated using confocal imaging and flow cytometry. Cells were treated with melatonin at concentrations of 0, 0.25, 0.5, or 1.0 mM. Fig 8A and 8B demonstrate a concentration-dependent, albeit modest, reduction in cellular fluorescence intensity, with the highest tested concentration of 1.0 mM melatonin inducing an 11.98% decrease. Flow cytometric analysis (Fig 8C and 8D) revealed a progressive decline in both the proportion of FOLH1-positive cells and the MFI with increasing melatonin concentrations. Furthermore, Transwell assays demonstrated dose-dependent suppression of LNCaP cell invasion and migration by melatonin (Fig 8E). Invasion and migration rates were quantified using ImageJ software and plotted in Fig 8F (n = 3, *p* < 0.05).

### 8.2. Circadian Rhythm Disruption Alters Melatonin Levels and FOLH1 Expression

To further validate the effects of melatonin on FOLH1 expression and tumor progression in vivo, we established xenograft models in nude mice subjected to distinct light-dark cycles and performed melatonin rhythm assessments. The experimental design comprised three groups of nude mice with varying photoperiods. LNCaP cells were implanted subcutaneously into these mice, and serum melatonin levels were measured at designated timepoints alongside tumor volume monitoring. Finally, we euthanized the mice and harvested tumor tissues at ZT16 (Zeitgeber Time 16). Altered light conditions significantly modulated melatonin levels: the normal circadian group exhibited robust diurnal melatonin fluctuations with higher overall levels, whereas the constant darkness group displayed reduced melatonin levels (though retaining residual rhythmicity). The disrupted rhythm group showed intermediate melatonin levels with abolished circadian patterns (Figs 8G, S5B).

Correspondingly, tumor growth was slowest in the circadian group, accompanied by the lowest FOLH1 fluorescence intensity. Conversely, the disrupted rhythm group demonstrated accelerated tumor growth and the highest FOLH1 fluorescence intensity (Fig 8H-8L). While limited by the small sample size (n = 3 per group), these preliminary findings suggest a potential association between endogenous melatonin levels, tumor growth, and FOLH1 expression dynamics.

## 9. Discussion

FOLH1, initially characterized by its aberrant elevation in prostate cancer, has evolved from a diagnostic biomarker to a clinically validated therapeutic target. The implementation of multiple FOLH1-targeted imaging modalities and therapeutic strategies in clinical practice underscores its significance in modern oncology [55–58]. Our study extends previous knowledge by comprehensively analyzing FOLH1 across diverse malignancies. Emerging evidence reveals that FOLH1 exhibits dysregulated expression across diverse malignancies and correlates with clinical prognosis [59, 60].Our study identifies melatonin as a potential FOLH1- modulating compound via an integrated AI-driven drug discovery pipeline, subsequently validated by cellular and animal experiments.

Expression profiling revealed significant FOLH1 upregulation in 16 malignancies (UCEC, LUAD, ESCA, STES, COAD, COADREAD, STAD, KIRC, LUSC, THCA, OV, PAAD, UCS, LAML, PCPG, ACC) beyond PRAD, while downregulation was observed in 10 cancer types (GBM, GBMLGG, LGG, BRCA, KIRP, LIHC, WT, SKCM, KICH, CHOL). Spatial distribution analysis indicates predominant FOLH1 elevation in epithelial-derived tumors and suppression in non-epithelial malignancies. This dichotomy likely reflects tissue-specific microenvironmental factors, particularly correlating with angiogenesis-related gene expression profiles and endothelial cell abundance [9]. While our computational analyses reveal compelling pan-cancer expression patterns, we acknowledge that the term “pan-cancer biomarker” should be interpreted with caution given our experimental validation focused primarily on prostate cancer models. In PRAD, FOLH1 overexpression associates with lymph node metastasis, suggesting its involvement in metastatic progression via enhanced tumor invasiveness. The age-dependent negative correlation implies potential hormonal or epigenetic regulatory influences [61].

By integrating multiple computational approaches for immune infiltration assessment (including ssGSEA, CIBERSORT, and MCP-counter), our study systematically characterizes FOLH1’s context-dependent immunomodulatory functions. This multi-algorithmic approach minimizes method-specific biases and strengthens the reliability of our findings regarding FOLH1’s dual roles in immune regulation. However, we acknowledge these analyses remain correlative and lack mechanistic depth regarding specific immune pathways such as macrophage polarization or Treg recruitment. In BRCA and LUAD, FOLH1 demonstrates significant positive correlations with immune cell infiltration (e.g., CD8^+^ T cells) and immunostimulatory genes (CD40LG, CXCL10), suggesting its potential to enhance anti-tumor immunity via immune cell recruitment. Paradoxically, FOLH1 exhibits negative correlations with MHC molecules in TGCT/LUSC, indicating possible immune evasion through antigen presentation suppression [62]. We note that the biological plausibility of these correlations in prostate cancer, known for sparse immune infiltration, requires further investigation. This tissue-specific immunoregulatory pattern positions FOLH1 as a potential biomarker for precision immunotherapy, though mechanistic elucidation requires single-cell sequencing and spatial transcriptomics.

UMAP analysis in PRAD localized FOLH1 enrichment predominantly within epithelial cells, implicating its role in metastasis via epithelial-mesenchymal transition (EMT) or cell adhesion molecule regulation. Despite associations with B-cell and macrophage infiltration, the negative correlation with immune infiltration scores suggests FOLH1 may promote immune evasion by recruiting immunosuppressive subsets (e.g., M2 macrophages) or secreting inhibitory cytokines (IL-10, TGF-β). This seemingly paradoxical phenomenon suggests that FOLH1 may drive tumor progression through a sophisticated “immune equilibrium disruption” mechanism rather than through simple immunosuppression [63]. We acknowledge that the single-cell analyses were limited to prostate cancer and future work should examine FOLH1 localization consistency across additional cancer types. Future studies employing spatial transcriptomics and cell-cell interaction network analyses will be essential to delineate the precise immunomodulatory mechanisms of FOLH1 in the prostate cancer microenvironment.

Our computational pipeline incorporated 28,241 ligands associated with prostate cancer-related target proteins, thereby facilitating a comprehensive machine learning analysis that effectively circumvented the critical limitation of sparse IC_50_ data specifically for FOLH1-interacting compounds. This transfer learning approach leverages the structural and biochemical similarities between FOLH1 and related proteins, significantly enhancing the predictive capacity of our models despite the limited availability of direct FOLH1 binding data. Among the evaluated models, the Extra Trees Regressor demonstrated the highest predictive accuracy and consistency, as evidenced by its superior performance in scatter plot distributions, fingerprint correlations, MAE, R² metrics, and Taylor diagram positioning. These results establish a reliable computational framework for identifying drug candidates with therapeutic potential against prostate cancer, with the Extra Trees Regressor emerging as the most effective tool for pIC_50_ prediction in this context.

Comprehensive characterization of the FOLH1 ligand chemical space revealed a non-uniform distribution of biological activity profiles. Ligand activity classification into low (pIC_50_ < 5), medium (5 ≤ pIC_50_ ≤ 7), and high (pIC_50_ > 7) categories. This categorization aligns with established criteria in drug discovery, where compounds with pIC_50_ > 7 (IC_50_ < 100 nM) are generally considered to possess potential therapeutic value due to their robust inhibitory activity at biologically relevant concentrations [64]. High-activity ligands (pIC_50_ > 7) exhibited not only numerical predominance but also remarkable structural diversity, as visualized through their extensive dispersion patterns in dimensionality-reduced t-SNE plots. This observation challenges the conventional assumption of structural convergence among high-affinity ligands and suggests multiple binding modes or interaction mechanisms with the FOLH1 protein. Medium-activity ligands clustered more tightly, while low-activity ligands were both scarce and sparsely distributed. Physicochemical descriptors further corroborated these trends: ligands predominantly exhibited drug-like properties (e.g., LogP < 5, MolWt < 500 Da, NumHDonors < 5), aligning with Lipinski’s Rule of Five, though exceptions (e.g., LogP > 5) were rare and structurally distinct. The K-means clustering, supported by pairwise relationship plots, successfully identified three clusters, each characterized by unique combinations of molecular features. These findings provide a detailed structural-activity landscape of the FOLH1 ligands, laying a foundation for subsequent drug candidate prioritization and optimization.

The integration of our ChemFusionSimilarity approach with the established Tanimoto similarity metric into a unified analytical framework enabled robust comparative assessment, revealing distinct yet complementary molecular similarity profiles. The superior performance of ChemFusionSimilarity demonstrates that our deep learning-based approach captures more nuanced molecular relationships than traditional fingerprint-based methods, particularly in identifying non-obvious structural analogues with potential therapeutic activity. The 3D density plot and joint distribution analysis underscored the consistency and linearity of both metrics, with ChemFusionSimilarity scores clustering at higher values (0.16–0.22) compared to Tanimoto scores (0.10–0.14). Violin plots further highlighted ChemFusionSimilarity’s broader and elevated distribution (0.125–0.250) relative to Tanimoto’s narrower range (0.075–0.150), suggesting enhanced sensitivity to molecular similarity. The predicted IC50 values, predominantly ranging from 300 to 400 nM with a minority below 150 nM, corroborate the model’s utility in identifying potent drug candidates. Collectively, these results affirm the ChemFusionSimilarity model’s superiority in capturing nuanced molecular relationships, offering a valuable tool for drug discovery efforts targeting FOLH1.

The UMAP-based screening reduced the initial DrugBank dataset from 12,457 drugs to 1,819 diverse candidates, of which 36 demonstrated high similarity (> 0.8) to FOLH1 ligands. Molecular docking of the top four candidates, Glutathione, Methotrexate, Melatonin, and Estrone, revealed binding affinities ranging from -9.670 to -11.078 kcal/mol, with Glutathione exhibiting the strongest interaction. The diverse interaction profiles, including hydrogen bonds, hydrophobic contacts, salt bridges, and pi-interactions, underscored the distinct binding mechanisms of each drug. These computational results serve as an exploratory foundation for identifying potential drug candidates. The predicted binding affinities of our top candidates (-9.6 to -11.0 kcal/mol) are mathematically comparable to previously published molecular docking analyses of established high-affinity PSMA ligands [65]. However, we emphasize that Glutathione’s mathematically ‘superior’ score over Melatonin is within the inherent error margins of the scoring function and does not guarantee superior biological efficacy. This analysis provides a foundation for further experimental validation and optimization of these candidates as therapeutic agents for prostate cancer.

Glutathione exhibited the highest binding affinity among the candidates, with a predicted binding free energy of -11.078 kcal/mol, surpassing Melatonin (-9.681 kcal/mol), Methotrexate (-10.236 kcal/mol), and Estrone (-9.670 kcal/mol). This strong affinity aligns with Glutathione’s high hydrogen bond donor and acceptor counts (6 HBD, 6 HBA), enabling extensive polar interactions with the target binding site, likely FOLH1 or a related protein (S4 File). Molecular docking analyses revealed that Glutathione’s tripeptide structure forms multiple hydrogen bonds and electrostatic interactions, contributing to its low binding energy. However, its cellular permeability was suboptimal, with a Cell Permeability Index (CPI) < 0.5, likely due to its high polarity (LogP -2.21) and molecular weight (307.08 Da), which may limit its bioavailability in cellular assays. Melatonin, in contrast, demonstrated a balanced profile, with a binding affinity of -9.681 kcal/mol and a CPI of 1.2, reflecting its moderate lipophilicity (LogP 0.15) and compact size (149.05 Da). Its simpler indole-based scaffold, with 2 HBD and 3 HBA, supports efficient binding while facilitating membrane crossing, a critical factor for intracellular targets. Methotrexate, a known FOLH1 inhibitor, showed comparable inhibition to Melatonin with both compounds exhibiting pIC_50_ values greater than 7. Methotrexate also showed a binding affinity of -10.236 kcal/mol and a CPI of 0.83. Its higher molecular weight (454.44 Da, outside Cluster 2’s range) and complex folate-like structure (5 HBD, 10 HBA, LogP 0.27) suggest a trade-off between potency and permeability, potentially limiting its efficacy in vivo. Estrone, with a binding affinity of -9.670 kcal/mol and a CPI of 1.0, exhibited promising activity but raised concerns due to its steroidal structure (LogP 3.82, 1 HBD, 2 HBA), which may induce hormonal side effects, such as estrogen receptor activation, complicating its therapeutic application. Melatonin emerged as the leading candidate due to its favorable efficacy-to-safety profile. Although Glutathione showed the highest binding affinity, we prioritized Melatonin for experimental validation due to its superior cell permeability and established safety profile. Additionally, Melatonin’s well-established clinical safety record, derived from decades of use as a sleep regulator, mitigates concerns about off-target effects. A critical limitation of our study is the absence of parallel experimental testing for our top computational hit (Glutathione) or the established FOLH1 inhibitor (Methotrexate). Testing these candidates alongside melatonin *in vitro* would have provided a much stronger, direct experimental validation of our computational ranking pipeline.

Unsupervised K-means clustering of the 36 FOLH1-matched drug candidates yielded three pharmacologically distinct chemical clusters, with clustering robustness validated through complementary statistical approaches. This optimal partitioning was further corroborated through bootstrap stability analysis, confirming the inherent structure within the chemical space of potential FOLH1-targeting drugs. PCA visualization and fingerprint analysis revealed cluster-specific distributions and structural patterns, with Cluster 2 (Glutathione, Melatonin, Estrone) and Cluster 3 (Methotrexate) encompassing high-activity drug compounds (pIC_50_ > 7). SAR analysis highlighted correlations between activity and physicochemical properties, such as smaller molecular weights and fewer hydrogen bond donors in Cluster 2, versus Methotrexate’s larger size and acceptor-rich profile in Cluster 3. The low ADR risk across candidates, combined with MCS insights (e.g., OH in high-activity drugs), reinforces the therapeutic promise of these compounds, particularly the top performers identified via molecular docking. These results provide a robust framework for prioritizing drug candidates for further development. While Glutathione demonstrated superior binding affinity, Melatonin emerged as the lead candidate due to its balanced profile of moderate binding affinity, favorable cell permeability, and established clinical safety. This strategic selection aligns with drug discovery practices that prioritize compounds with optimal pharmacological properties over those with maximal binding affinity but poor drug-like characteristics.

It is crucial to recognize that FOLH1expression is not a static biological marker; rather, it is highly dynamic and subject to complex regulatory mechanisms within the tumor microenvironment. Our computational and experimental findings regarding FOLH1 modulation should be interpreted within this dynamic framework. Compounds like melatonin may not simply bind a static target but could potentially intersect with broader neuroendocrine or stress-response signaling pathways that indirectly influence FOLH1 expression levels and tumor cellular states.

Our in vitro functional validation studies demonstrated that melatonin treatment attenuated FOLH1 expression in the LNCaP prostate cancer cell line. However, we explicitly acknowledge the preliminary nature of these findings. The observed reduction in FOLH1 signal was modest (~12%) and required a relatively high, supra-physiological melatonin concentration (1.0 mM). Furthermore, because our experimental scope was limited to a single cell line, the generalizability of this effect across other prostate cancer models remains to be determined. Mechanistically, we did not investigate whether this suppression occurs at the transcriptional or post-translational level; thus, definitive mechanistic conclusions cannot be drawn from the current data. While previous literature suggests melatonin may influence transcription factors or epigenetic modifications, future studies incorporating additional cell lines and precise molecular assays (e.g., RT-qPCR, Western blot) are strictly required to elucidate the specific regulatory mechanism behind this FOLH1 suppression. Transwell assays confirmed melatonin’s inhibitory effects on LNCaP cell migration/invasion, potentially via downregulation of matrix metalloproteinases (MMPs) or integrin signaling modulation [66]. However, the causal relationship between FOLH1 suppression and inhibited migration/invasion requires validation through FOLH1 knockdown/overexpression experiments.

Our xenograft studies in immunocompromised mouse models revealed that experimentally induced circadian rhythm disruption significantly altered endogenous melatonin levels, which strongly correlated with increased FOLH1 expression and accelerated tumor growth. We deeply acknowledge the limitation of the small sample size (n = 3 per group) which critically reduces statistical power and precludes any strong causal conclusions. These in vivo findings must be considered strictly preliminary, and validation in appropriately powered, larger animal cohorts is required to determine whether this relationship is truly causal or merely correlative. These preliminary observations highlight a potential correlation among circadian rhythms, melatonin levels, and FOLH1 expression, with possible implications for both the timing of diagnostic imaging and therapeutic interventions in prostate cancer patients [67]. Potential mechanisms involve Warburg effect modulation, lipid signaling, proliferation control [68], or circadian gene regulation (CLOCK/BMAL1) of FOLH1 transcription [69]. Circadian disruption (e.g., shift work/sleep disorders) correlates with elevated prostate cancer risk [70,71], with this study providing first experimental evidence for melatonin-FOLH1 pathway mediation. These findings suggest temporal considerations for FOLH1-targeted imaging/therapy efficacy. However, the detailed mechanism of this pathway is not yet fully understood, and additional molecular and cellular experiments may be necessary for further research.

Our study presents several notable strengths: 1) the comprehensive pan-cancer analysis of FOLH1 expression across 33 cancer types using multiple independent datasets (TCGA, GTEx, UCSC Xena, HPA); 2) the development of a novel computational pipeline integrating multiple molecular representation techniques with deep learning approaches; 3) the successful identification and experimental validation of melatonin as an agent capable of suppressing FOLH1 expression; and 4) the discovery of a previously unrecognized circadian regulation of FOLH1 expression with potential clinical implications. Nevertheless, several limitations should be acknowledged. First, our in vitro experimental validation is preliminary, being limited to a single prostate cancer cell line (LNCaP) and demonstrating only a modest reduction in FOLH1 expression at high melatonin concentrations. Expanded experimental validation across diverse cell lines and dedicated mechanistic molecular assays are required. Second, the retrospective nature of our survival analyses limits causal interpretations regarding FOLH1’s prognostic significance. Third, the precise mechanism of melatonin-mediated FOLH1 suppression remains unestablished. Our molecular docking analysis is strictly exploratory; while it predicts favorable spatial interactions, docking alone does not establish binding specificity or functional inhibition of the target. Claims of high-affinity targeting cannot be confirmed solely through in silico methods. Future biochemical and enzymatic validation assays—such as NAALADase enzymatic inhibition assays or Surface Plasmon Resonance (SPR)—are absolutely required to substantiate whether melatonin acts as a direct, specific inhibitor of the FOLH1 protein. Fourth, the animal studies were underpowered, and future work should include proper sample size justification and larger group sizes. Fifth, our experimental follow-up of the computational predictions is extremely limited. Multiple candidate drugs were identified in silico, yet only melatonin was evaluated in vitro and in vivo. This lack of experimental comparison with other top candidates restricts our ability to definitively validate the accuracy of our computational prioritization. Finally, the potential interactions between melatonin and current FOLH1-targeted therapies warrant investigation to determine optimal combination strategies.

In summary, our study provides a comprehensive characterization of FOLH1 expression patterns across diverse malignancies and demonstrates its multifaceted roles in cancer progression and immune modulation. Through an innovative computational drug discovery pipeline integrating machine learning, molecular docking, and similarity analyses, we identified melatonin as a promising candidate predicted to interact with FOLH1. Subsequent experimental validation confirmed melatonin’s ability to suppress FOLH1 expression and inhibit prostate cancer cell migration and invasion, while also uncovering a novel circadian regulatory mechanism. These findings establish melatonin as an exploratory candidate for further development as a potential FOLH1-modulating compound, though substantial mechanistic work—including direct binding assays— remains to fully characterize its mode of action and therapeutic potential. Future studies should evaluate the efficacy of melatonin supplementation in enhancing responses to existing FOLH1-targeted therapies and ‌‌investigate chronotherapeutic approaches to optimize treatment outcomes in prostate cancer patients.

## Supporting information

S1 Fig Analysis of FOLH1 Expression Across Single-cell Types and Stratified by Lymph Node Metastasis Status. (A) Summary of normalized single-cell FOLH1 RNA expression (nTPM) across all cell types. Color-coding corresponds to cell type classification. (B) Pan-cancer analysis of FOLH1 expression stratified by lymph node metastasis status. Pairwise comparative analysis was performed using unpaired Student’s t-test, while multi-group comparisons were assessed via ANOVA. *p < 0.05, **p < 0.01, ***p < 0.001.
(TIF)

S2 Fig Algorithm-based correlations between FOLH1 expression and pan-cancer immune cell infiltration. (A-E) correspond to CIBERSORT, EPIC, MCP-Counter, QUANTISEQ, and TIMER algorithms, respectively.
(TIF)

S3 Fig Correlations between FOLH1 and immune-modulating genes across 44 cancer types (*p < 0.05). (TIF)

S4 Fig Model Performance, Molecular Similarity Metrics, and Structure-Activity Relationships in Drug Compound Activity Prediction (A) Perturbation Analysis of Machine Learning Models. The robustness of models was assessed through controlled input perturbations. The plots display changes in Mean Absolute Error (MAE) and coefficient of determination (R²) as perturbation intensity increases. (B) 3D density plot illustrating the relationship between ChemFusionSimilarity and Tanimoto similarity scores. (C) Joint distribution plot depicting the strong linear correlation between ChemFusionSimilarity and Tanimoto similarity with marginal distributions confirming concentration of scores within characteristic ranges. (D) Violin plots comparing distributional characteristics of both similarity metrics, revealing ChemFusionSimilarity’s broader range and consistently higher values compared to Tanimoto similarity’s narrower distribution. (E) Distribution of predicted IC_50_ values using the ChemFusionSimilarity model, showing majority of predictions between 300–400 nM (moderate to high potency) and a smaller subset below 150 nM (exceptionally high activity). (F) Relationship Between Topological Polar Surface Area and Activity. Scatter plot displaying the correlation between Topological Polar Surface Area (TPSA, Å²) and compound activity (pIC_50_). (G) Impact of Molecular Descriptors on Activity. Bar chart illustrating the relative importance of molecular descriptors in predicting compound activity (pIC_50_) based on coefficient magnitudes.
(TIF)

S5 Fig (A) CCK-8 assay evaluating melatonin’s effect on LNCaP cell viability. (B) Dynamic changes in melatonin levels under distinct circadian rhythm conditions.
(TIF)

S1 File FOLH1 Ligand Dataset with Structural and Physicochemical Properties. Comprehensive dataset of FOLH1 ligands with structural and physicochemical characterization. This dataset contains 420 FOLH1 ligands with their corresponding pIC_50_ values, calculated molecular descriptors, structural and physicochemical properties.
(CSV)

S2 File Combined ChemFusionSimilarity and Tanimoto Similarity Dataset. Comparative analysis of ChemFusionSimilarity and Tanimoto similarity metrics. This dataset integrates similarity scores from both the deep learning-based ChemFusionSimilarity model and the traditional Tanimoto similarity metric for the FOLH1 ligands.
(CSV)

S3 File Structurally Diverse Drug Candidate Dataset. Structurally diverse drug candidates selected from DrugBank database. This dataset contains 1,819 drug compounds refined from an initial set of 12,457 DrugBank entries using Uniform Manifold Approximation and Projection (UMAP) dimensionality reduction. The selection process prioritized molecular diversity by eliminating structurally redundant compounds, resulting in a comprehensive collection of candidates for similarity matching with FOLH1 ligands.
(CSV)

S4 File High-Similarity Drug Candidates for FOLH1 Targeting. Drug candidates with high similarity to known FOLH1 ligands. This dataset presents 36 drug compounds from the refined candidate pool that exhibited similarity scores exceeding 0.8 with known FOLH1 ligands, as determined using ChemFusionSimilarity. These compounds represent promising candidates for potential FOLH1 modulation based on their strong structural resemblance to established FOLH1 ligands.
(CSV)

S1 Table Comparison of Cross-Validation and Test Set Performance Metrics for Machine Learning Models in Effective Drug Compound Activity Prediction. (DOCX)
