# Supplementary figures and images for: Integrated computational and experimental analysis explores FOLH1 expression patterns across cancers and nominates melatonin as a potential modulator in prostate cancer models
Source: PLoS Comput Biol. 2026 May 22;22(5):e1014315. doi: 10.1371/journal.pcbi.1014315 (PMC13218620; doi:10.1371/journal.pcbi.1014315)

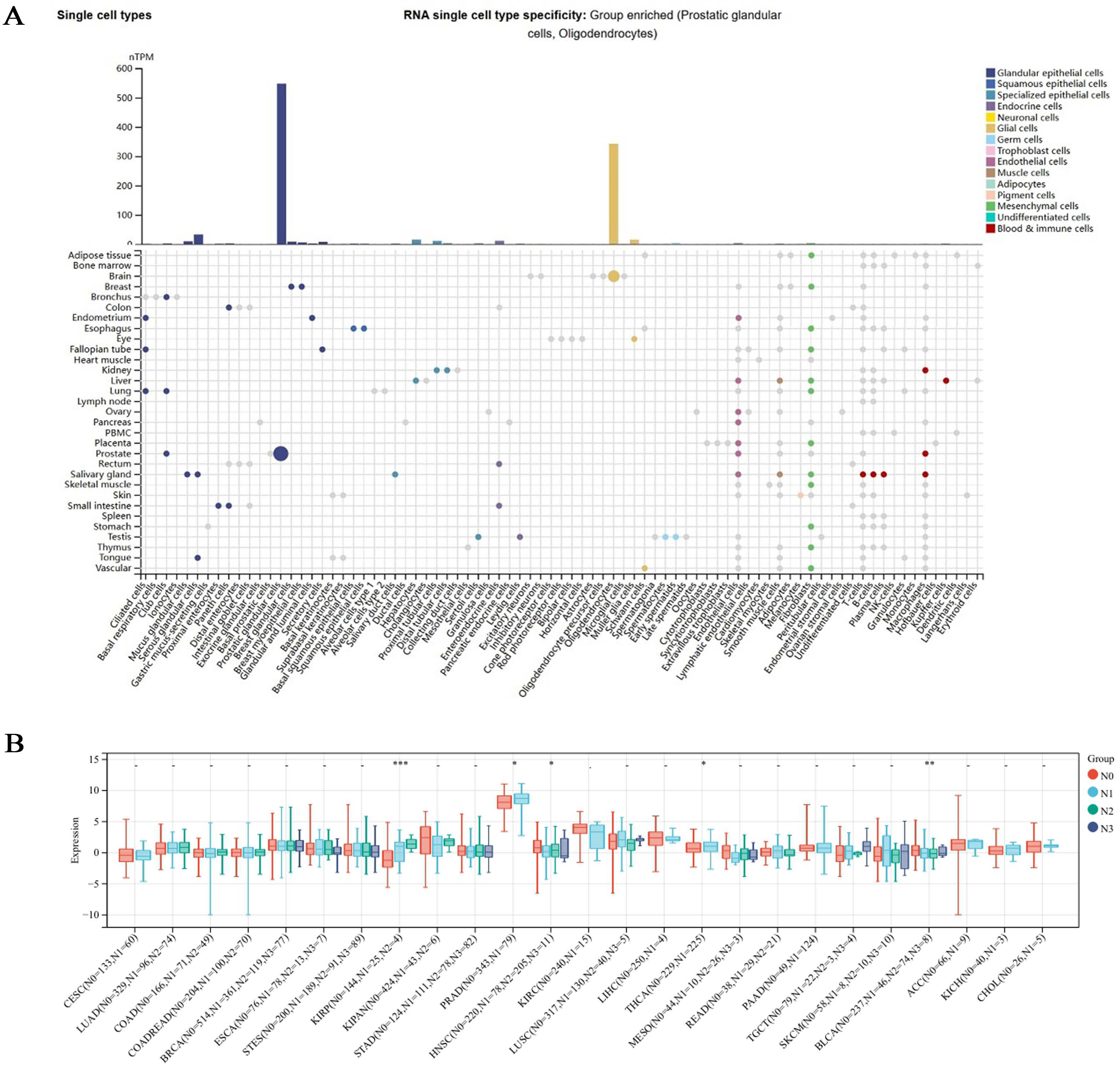

Supplement: S1 Fig — (A) Summary of normalized single-cell FOLH1 RNA expression (nTPM) across all cell types. Color-coding corresponds to cell type classification. (B) Pan-cancer analysis of FOLH1 expression stratified by lymph node metastasis status. Pairwise comparative analysis was performed using unpaired Student’s t-test, while multi-group comparisons were assessed via ANOVA. *p < 0.05, **p < 0.01, ***p < 0.001. (TIF) [file pcbi.1014315.s001.tif]
