# Supplementary material for: Integrated computational and experimental analysis explores FOLH1 expression patterns across cancers and nominates melatonin as a potential modulator in prostate cancer models
Source: PLoS Comput Biol. 2026 May 22;22(5):e1014315. doi: 10.1371/journal.pcbi.1014315 (PMC13218620; doi:10.1371/journal.pcbi.1014315)
Supplement: S1 Table — (DOCX) [file pcbi.1014315.s010.docx]

Supplementary Table S1. Comparison of Cross-Validation and Test Set Performance Metrics for Machine Learning Models in Effective Drug Compound Activity Prediction

|  | Cross-Validation Results | | | | Test Results | | | |
| --- | --- | --- | --- | --- | --- | --- | --- | --- |
|  | Accuracy^a^ | Precision^b^ | Recall^c^ | F1-Score^d^ | Accuracy^e^ | Precision^f^ | Recall^g^ | F1-Score^h^ |
| Ridge^i^ | 0.9217 | 0.9095 | 0.9012 | 0.9038 | 0.8048 | 0.8387 | 0.7667 | 0.7908 |
| LinearSVR^j^ | 0.7471 | 0.7596 | 0.7624 | 0.7568 | 0.7714 | 0.7832 | 0.7619 | 0.7699 |
| ExtraTrees^k^ | 0.9452 | 0.9374 | 0.9429 | 0.9351 | 0.9126 | 0.9074 | 0.9206 | 0.9141 |
| LGBMRegressor^l^ | 0.8905 | 0.9059 | 0.8714 | 0.8883 | 0.7861 | 0.7832 | 0.7124 | 0.7161 |
| XGBoost^m^ | 0.9429 | 0.9209 | 0.9048 | 0.9382 | 0.7381 | 0.7687 | 0.7381 | 0.7388 |
| RandomForest^n^ | 0.9262 | 0.9382 | 0.9138 | 0.9269 | 0.7595 | 0.7887 | 0.7524 | 0.7602 |
| CNN^o^ | 0.8476 | 0.8286 | 0.8125 | 0.8354 | 0.7048 | 0.7278 | 0.7063 | 0.7029 |
| GNN^p^ | 0.7269 | 0.7667 | 0.7586 | 0.7206 | 0.6381 | 0.6333 | 0.6386 | 0.6359 |

The performance of various machine learning models evaluated for drug screening. The metrics include ^a^Accuracy, overall correctness of predictions on the cross-validation sets; ^b^Precision, proportion of correctly predicted positive pIC_50_ out of all predicted positive pIC_50_ on the cross-validation sets; ^c^Recall, proportion of correctly predicted positive pIC_50_ out of all actual positive pIC_50_ on the cross-validation sets; ^d^F1-Score, the harmonic mean of precision and recall on the cross-validation sets. The Cross-Validation Results were obtained through a 5-fold cross-validation procedure, providing a robust estimate of the model's generalization ability. The metrics on the independent test set are ^e^Accuracy, overall correctness of predictions on the test set; ^f^Precision, proportion of correctly predicted positive pIC_50_ out of all predicted positive pIC_50_ on the test set; ^g^Recall, proportion of correctly predicted positive pIC_50_ out of all actual positive pIC_50_ on the test set; and ^h^F1-Score, the harmonic mean of precision and recall on the test set. The models evaluated are ^i^Ridge, Ridge Regression; ^j^LinearSVR, Linear Support Vector Regression; ^k^ExtraTrees, Extra Trees Regressor; ^l^LGBMRegressor, Light Gradient Boosting Machine Regressor; ^m^XGBoost, Extreme Gradient Boosting; ^n^RandomForest, Random Forest; ^o^CNN, Convolutional Neural Network; and ^p^GNN, Graph Neural Network.

Supplementary Table S2: Final Hyperparameter Configurations for Machine Learning Models in Drug Compound Activity Prediction

| Model | Hyperparameter | Value | Tuned or Default | Notes |
| --- | --- | --- | --- | --- |
| Ridge^a^ | alpha | 1.0 | Tuned | Regularization strength, tuned via grid search |
|  | fit_intercept | True | Default | Default setting in scikit-learn (v1.5.2) |
|  | max_iter | 1000 | Default | Maximum iterations for convergence |
| LinearSVC^b^ | C | 0.1 | Tuned | Regularization parameter, tuned via grid search |
|  | epsilon | 0.01 | Tuned | Epsilon-tube for loss function, tuned via grid search. |
|  | fit_intercept | True | Default | Default setting in scikit-learn (v1.5.2) |
|  | max_iter | 1000 | Default | Maximum iterations for optimization |
| ExtraTrees^c^ | n_estimators | 200 | Tuned | Number of trees, tuned via grid search |
|  | max_depth | None | Default | No maximum depth restriction (full tree growth) |
|  | min_samples_split | 2 | Default | Minimum samples required to split a node |
|  | min_samples_leaf | 1 | Default | Minimum samples required at a leaf node |
|  | max_features | auto | Tuned | Number of features to consider for splits (sqrt of total features) |
| LGBM Regressor^d^ | learning_rate | 0.05 | Tuned | Learning rate, tuned via grid search |
|  | n_estimators | 300 | Tuned | Number of boosting iterations, tuned via grid search |
|  | max_depth | 7 | Tuned | Maximum tree depth, tuned via grid search |
|  | num_leaves | 31 | Tuned | Maximum number of leaves per tree, tuned via grid search |
|  | min_child_samples | 20 | Default | Minimum number of samples in a leaf (LightGBM v4.5.0) |
|  | reg_lambda | 0.0 | Default | L2 regularization term on weights |
| XGBoost^e^ | learning_rate | 0.1 | Tuned | Learning rate (eta), tuned via grid search |
|  | n_estimators | 200 | Tuned | Number of boosting iterations, tuned via grid search |
|  | max_depth | 6 | Tuned | Maximum tree depth, tuned via grid search |
|  | lambda | 1.0 | Default | L2 regularization term on weights (XGBoost v3.0.1) |
|  | alpha | 0.0 | Default | L1 regularization term on weights |
|  | subsample | 0.8 | Tuned | Fraction of samples used per tree, tuned via grid search |
| GNN^f^ | num_layers | 3 | Tuned | Number of GNN layers, tuned via grid search. |
|  | hidden_dim | 128 | Tuned | Dimension of hidden node features, tuned via grid search |
|  | aggregation | mean | Tuned | Aggregation function for message passing |
|  | learning_rate | 0.001 | Tuned | Learning rate for Adam optimizer, tuned via grid search |
|  | dropout | 0.2 | Tuned | Dropout rate for regularization, tuned via grid search |
|  | batch_size | 64 | Tuned | Batch size for training, tuned via grid search |
| CNN^g^ | num_conv_layers | 2 | Tuned | Number of convolutional layers, tuned via grid search |
|  | filters | [64, 128] | Tuned | Number of filters per layer, tuned via grid search |
|  | kernel_size | 3 | Tuned | Size of convolutional kernels, tuned via grid search |
|  | pooling | max | Tuned | Pooling strategy (max pooling) |
|  | learning_rate | 0.001 | Tuned | Learning rate for Adam optimizer, tuned via grid search |
|  | dropout | 0.3 | Tuned | Dropout rate for dense layers, tuned via grid search |
|  | batch_size | 32 | Tuned | Batch size for training, tuned via grid search |
| Random Forests^h^ | n_estimators | 150 | Tuned | Number of trees, tuned via grid search |
|  | max_depth | None | Default | No maximum depth restriction (full tree growth) |
|  | min_samples_split | 2 | Default | Minimum samples required to split a node |
|  | min_samples_leaf | 1 | Default | Minimum samples required at a leaf node |
|  | max_features | auto | Tuned | Number of features to consider for splits (sqrt of total features) |

The final hyperparameters used for Each model in the reported results, including both tuned parameters, optimized via grid search during 5-fold cross-validation, and default parameters. Models were implemented using standard machine learning libraries, with specific versions noted where applicable. All models were trained on the feature matrix derived from the training set.The models evaluated are ^a^Ridge, Ridge Regression; ^b^LinearSVR, Linear Support Vector Regression; ^c^ExtraTrees, Extra Trees Regressor; ^d^LGBMRegressor, Light Gradient Boosting Machine Regressor; ^e^XGBoost, Extreme Gradient Boosting; ^f^GNN, Graph Neural Network; ^g^CNN, Convolutional Neural Network; and ^h^RandomForest, Random Forest.
